# A predictive in vitro risk assessment platform for pro-arrhythmic toxicity using human 3D cardiac microtissues

**DOI:** 10.1038/s41598-021-89478-9

**Published:** 2021-05-13

**Authors:** Celinda M. Kofron, Tae Yun Kim, Fabiola Munarin, Arvin H. Soepriatna, Rajeev J. Kant, Ulrike Mende, Bum-Rak Choi, Kareen L. K. Coulombe

**Affiliations:** 1 Center for Biomedical Engineering, School of Engineering, Brown University, Providence, RI USA; 2 Cardiovascular Research Center, Cardiovascular Institute, Rhode Island Hospital and Alpert Medical School of Brown University, Providence, RI USA

**Keywords:** Biomedical engineering, Cardiovascular models, Biotechnology, Tissue engineering, Biological models, Biological techniques, Electrophysiology, Extracellular recording

## Abstract

Cardiotoxicity of pharmaceutical drugs, industrial chemicals, and environmental toxicants can be severe, even life threatening, which necessitates a thorough evaluation of the human response to chemical compounds. Predicting risks for arrhythmia and sudden cardiac death accurately is critical for defining safety profiles. Currently available approaches have limitations including a focus on single select ion channels, the use of non-human species in vitro and in vivo, and limited direct physiological translation. We have advanced the robustness and reproducibility of in vitro platforms for assessing pro-arrhythmic cardiotoxicity using human induced pluripotent stem cell-derived cardiomyocytes and human cardiac fibroblasts in 3-dimensional microtissues. Using automated algorithms and statistical analyses of eight comprehensive evaluation metrics of cardiac action potentials, we demonstrate that tissue-engineered human cardiac microtissues respond appropriately to physiological stimuli and effectively differentiate between high-risk and low-risk compounds exhibiting blockade of the hERG channel (E4031 and ranolazine, respectively). Further, we show that the environmental endocrine disrupting chemical bisphenol-A (BPA) causes acute and sensitive disruption of human action potentials in the nanomolar range. Thus, this novel human 3D in vitro pro-arrhythmic risk assessment platform addresses critical needs in cardiotoxicity testing for both environmental and pharmaceutical compounds and can be leveraged to establish safe human exposure levels.

## Introduction

Cardiotoxicity in response to drugs, chemicals, and other environmental toxicants entails both structural and functional changes, and potentially lethal pro-arrhythmic effects continue to be of particular concern. Notably, drug-induced QT prolongation and Torsades de Pointes (TdP), fatal arrhythmias associated with sudden cardiac death, are the most common cause of withdrawal or restriction of the use of drugs on the market^1–7^ despite intense preclinical screening in silico, in vitro, and in animal models. Additionally, the World Health Organization (WHO) estimates that environmental toxicants such as pesticides and industrial chemicals may evoke up to 23% of cardiovascular diseases globally^8^, but the Environmental Protection Agency (EPA) still has little effort focused on cardiotoxicity, even with the current state-of-the-art computational approaches to chemical safety evaluation of Toxicity Forecaster (ToxCast) and Toxicity Testing in the 21st Century (Tox21). The high prevalence of drug- and chemical-induced pro-arrhythmic cardiotoxicity that can increase the risk for stroke, heart attack, heart failure, and sudden cardiac death^9^. point to a continued need for more reliable and robust risk assessment efforts to predict cardiotoxic effects with high sensitivity.

Existing pro-arrhythmia assays have a few key limitations. Animal models are complex and have limited predictability of human biological responses due to species-specific differences in ion channel expression that affect depolarization and repolarization kinetics of cardiac action potentials (APs) as well as differential sensitivity to pharmacological agents^10,11^. Human ether-a-go-go (hERG) channel blockade in vitro and corrected QT interval (QTc) prolongation in patients^12,13^. have long been used as effective and selective ‘biomarkers’ in compound screening to identify pro-arrhythmic risks that cause TdP. However, it is now recognized that a ‘hERG-centric’ approach does not capture all drug-induced cardiac arrhythmia mechanisms^14–18^ and can also lead to unnecessary discontinuation of compounds from development due to false positive outcomes despite clinical utility below an arrhythmogenic dose^19–21^. More recently developed in vitro cell-based models are often limited to 2D monolayers and lack sufficient myocardial cell–cell interactions, including heterotypic interactions between cardiomyocytes and cardiac fibroblasts, which modulate excitation–contraction in cardiomyocytes and thus affect the arrhythmogenic phenotype^22^.

New initiatives in toxicity screening specifically call for the development and implementation of non-animal approaches to assess potential hazards associated with acute and chronic exposures to industrial chemicals and medical products. Groups such as the Interagency Coordinating Committee on the Validation of Alternative Methods (ICCVAM) were assembled in response to new laws across the globe and point to a strong value in platforms based on a mechanistic understanding of toxicity^23,24^. Continued development of new approach methodologies (NAMs) to enhance predictive capabilities for prioritization, hazard screening, and risk assessment of cardiotoxicity is thus warranted^25^.

The inter-agency Comprehensive In Vitro Pro-arrhythmia Assay (CiPA) initiative has identified necessary goals for advancing cardiotoxicity testing: defining drug effects on seven human cardiac currents with automated patch clamping, using in silico models to define pro-arrhythmia risk metrics, verifying effects on cardiomyocytes derived from human induced pluripotent stem cells cultured in 2-dimensional monolayers, and validating with human clinical data for in vitro to in vivo extrapolation (IVIVE)^26,27^. Human induced pluripotent stem cell (hiPSC)-derived cardiomyocytes (CMs) are regarded as the future of NAMs for cardiotoxicity evaluation for their appropriate physiology, including proper human ion channel expression, AP shape, and rhythmic contractions^28–31^. HiPSC-CMs show great promise in detecting drug-induced pro-arrhythmic effects based on electrophysiological responses^32^. One complication in the field arises from cell-to-cell and batch-to-batch variability of hiPSC-CMs, which limits comparison within a narrow and defined time within a same batch. In addition, hiPSC-CM-based models built from traditional 2D culture systems do not recapitulate the cellular architecture of native tissue. Scaffold-supported and self-assembled cultures provide a 3D environment for more mature CM function^33,34^, and microtissues maximize cell-to-cell connectivity while allowing for easy visual assessment of the action potential (AP) and calcium transients through fluorescent reporters^35,36^.

Several groups are leveraging the utility of 3D cardiac microtissues for toxicant screening^22^, focusing on cell viability^37,38^, gene expression^39^, and mechanical outcomes such as force generation and contractility^22,40–44^, but not yet on diverse pro-arrhythmic metrics such as AP, calcium transient, and conduction velocity. Some 3D platforms that focus on arrhythmia have limited throughput (e.g., confocal microscopy^34^. or single microelectrode recording^42^) and focus on spontaneous beating frequency^45^, intracellular calcium flux recordings to infer AP changes^46^, or multi-electrode array recordings of the extracellular field potentials that can have inter test-site differences^47–51^. Many of these studies do not report characterization of reproducibility in light of batch-to-batch variability that is inherent to stem maintenance, differentiation, and purification protocols^52,53^. Important nuances of excitation that these approaches are not designed to capture and assess include non-uniform reduced excitation within a single microtissue; waveform changes such as upstroke, plateau level, or recovery rate of APs to predict ion channel blockade or Ca^2^.^+^ handling anomalies; APD triangulation related to intra-phase prolongation; and pro-arrhythmic triggered activity classification based on mechanism such as early- (EADs) and delayed-afterdepolarizations (DADs)^54,55^.

In this study, our objective was to fill these gaps and thereby advance predictive preclinical models of human drug-induced pro-arrhythmic risk using hiPSC-CMs and human cardiac fibroblasts (hCFs) in 3D self-assembled engineered microtissues. Utilizing optical mapping of voltage sensitive dyes, an established approach to evaluate the cardiac AP^54^, we developed an automated analysis pipeline to produce eight comprehensive evaluation metrics of the AP with reduced bias and increased throughput for risk assessment that capture pro-arrhythmic activity. To establish the fitness of this NAM to predict arrhythmogenesis, we thoroughly evaluated variability of our platform including beat-to-beat, microtissue-to-microtissue, mold-to-mold, and batch-to-batch for comprehensive statistical analysis of action potentials in response to test compounds. We validated our model with hERG channel blockers classified as high-risk and low-risk for TdP by CiPA^56,57^. We also evaluated a compound with poorly described human arrhythmic cardiotoxicity, bisphenol A (BPA), an industrial chemical and environmental toxicant classified as an endocrine disrupting chemical. BPA was found to alter excitation with nanomolar sensitivity, showing a dose-dependent reduction in AP duration in hiPSC-CMs, suggesting that our model is sensitive, selective, and fit for the purpose of human cardiac risk assessment.

## Results

### Development of 3D human cardiac microtissues using hiPSC-CMs and hCFs

**Figure 1:**
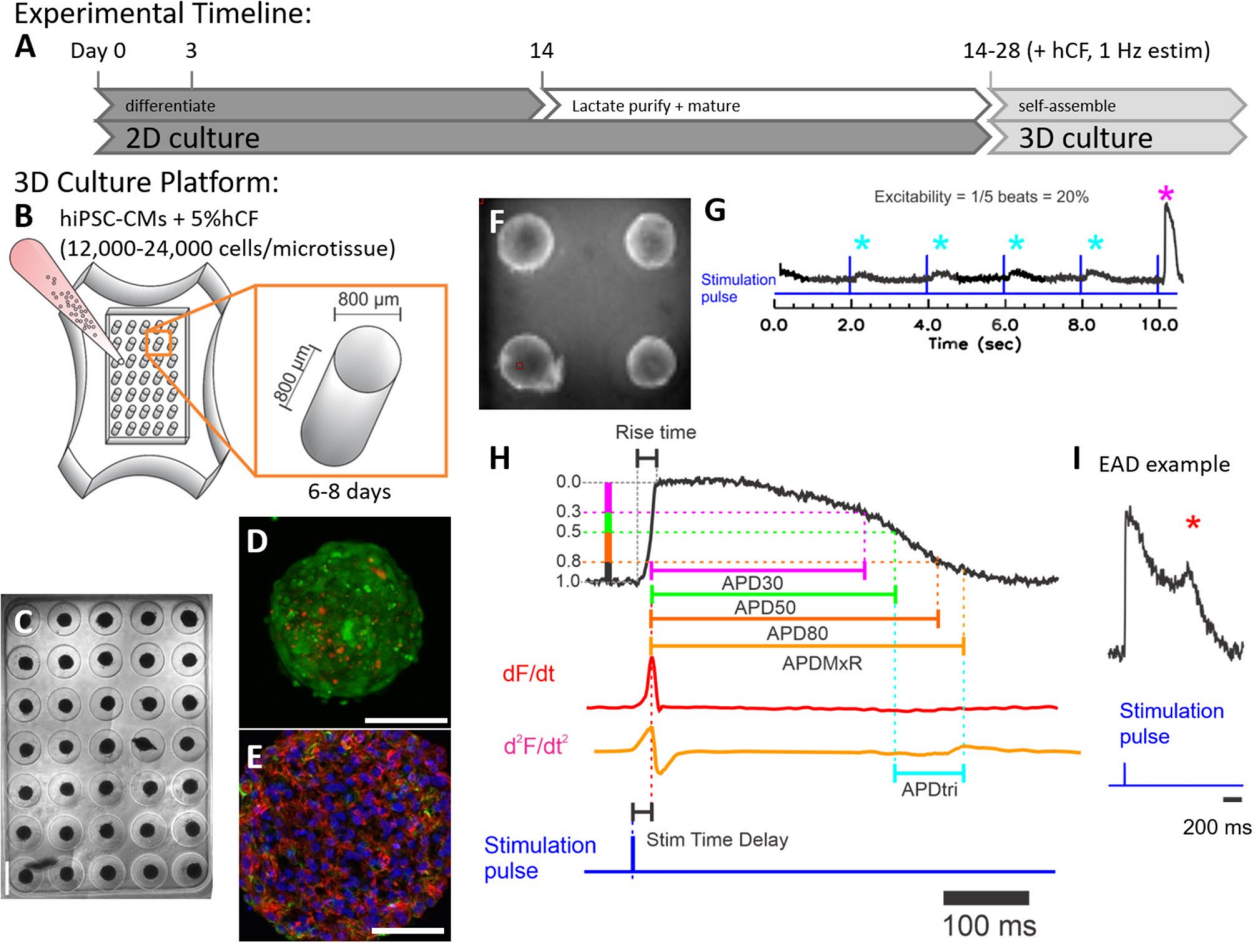
Differentiation of hiPSC-CM, formation of cardiac microtissues, and definitions of metrics. (**A**) Timeline shows cardiomyocyte differentiation from human-induced pluripotent stem cells (hiPSCs) in high density 2D culture. Cardiac directed differentiation was achieved with Wnt activation at day 1 and inhibition at day 3 (see “Methods”). Cardiac phenotype, visually confirmed by beating cells, appeared between days 8 and 12. Cardiomyocytes differentiated from hiPSCs were used for the production of microtissues or were further purified with a lactate-based metabolic selection protocol. Microtissues self-assembled in microwells after 14–28 days of differentiation of hiPSC-CMs (i.e., without or with lactate purification) and addition of human cardiac fibroblasts (hCFs), and they were cultured in the presence of 1 Hz electrical field stimulation (estim). (**B**) Schematic of three-dimensional (3D) cardiac microtissue generation shows non-adhesive agarose gels with cylindrical recesses with hemispherical bottoms that guide self-assembly. Cardiac microtissues were cultured for 6–8 days with 1 Hz pacing. (**C**) Phase contrast image shows consistent spherical microtissue formation after 5 days of 3D culture in all 35 microwells. Scale bar, 800 μm. (**D**) Confocal image shows a representative cardiac tissue with hiPSC-CM (green) and hCF (red) stained with CellTracker dyes. Scale bar, 200 μm. (**E**) Confocal image shows a representative cardiac troponin I (red), vimentin (green), and DAPI stained cryosection (10 μm thick) of a microtissue fixed after 7 days in 3D culture. Scale bar, 50 μm. (**F**) Fluorescence image of microtissues at 3.2 × magnification was obtained during optical mapping. Typically, the action potentials (APs) from 4–9 microtissues were recorded simultaneously. (**G**-**I**) Schematics of the AP metrics of that were defined (with units) as: (**G**) “excitability” (%) measured from the percentage of captured APs during 10 s duration of recording with 2 s pacing cycle length, (**H**) “stimulation time delay” (ms; stim delay) between stimulation pulse and evoked AP upstroke (dF/dt_max_), “rise time” (ms) of AP, “AP duration” (ms) to 30%, 50%, and 80% repolarization (APD_30_, APD_50_, APD_80_), “APD to the maximum repolarization rate” (ms; APD_MxR_) defined as time between AP upstroke and the end of rapid repolarization marked by d^2^F/dt^2^_max_, “APD triangulation” (ms; APD_tri_) defined as APD_MxR_—APD_50_, and (**I**) occurrence of “early afterdepolarization” (EAD) reported as (%) of microtissues showing EADs.

To develop a predictive human cardiotoxicity model to specifically address risk of cardiac arrhythmias, we adapted a scaffold-free cardiac microtissue model we previously developed with rat primary cardiac cells^35,36^. Here, we used hiPSC-CMs without or with lactate-based purification (hiPSC-CM_LP_; Fig. 1A) and primary normal adult human cardiac fibroblasts (hCFs) to generate 35 self-assembled microtissues per non-adhesive agarose gel containing cylindrical microwells with hemispherical bottoms (Fig. 1B). The structural and functional features of these microtissues were assessed with live cell imaging (Fig. 1C,E), immunohistochemistry and confocal imaging (Fig. 1D), and optical mapping (Fig. 1F,G). Consistent and reliable formation of 35 viable spherical microtissues per well was achieved (Fig. 1C, Movie S1) with the addition of 5% hCF to the cultures, increasing the reliability of self-assembly to incorporate all cells in a microwell (data not shown). Microtissue size varied with plating density. For microtissues generated from lactate-purified hiPSC-CMs, a plating density of 13,500 cells per microtissue (95% hiPSC-CM_LP_ and 5% hCF) yielded spherical microtissues 359.4 ± 32.4 μm in diameter after 1d in 3D culture that compacted to 290.4 ± 29.5 μm in diameter after 5d. A plating density of 15,000 cells per microtissue (95% hiPSC-CM_LP_ and 5% hCF) yielded spherical microtissues 400.4 ± 24.5 μm in diameter after 1d and 329.4 ± 17.5 μm after 5d. Use of CellTracker dyes to label hiPSC-CM (green) and hCF (red) prior to assembly as well as immunostaining with antibodies against cardiac troponin I (cTnI) and vimentin (vim) after fixation and sectioning confirmed that hiPSC-CMs and hCFs self-assembled to be highly interspersed (Fig. 1D,E), consistent with our prior work in rat cardiac microtissues^35,36^.

### Electrophysiological characterization of 3D cardiac microtissues

In order to evaluate excitability and sensitivity to pro-arrhythmic toxicants, APs were recorded from the human cardiac 3D microtissues with voltage sensitive dyes (Fig. 1F). Cardiac microtissues were paced at 2 s basic cycle length (0.5 Hz) and APs were recorded for 8–10 s. Human cardiac 3D microtissues showed robust fluorescence changes tracing V_m_ signal of APs during spontaneous and paced beats (Movie S2). The spontaneous rate could not be reliably measured because spontaneous action potentials were very rare and often only one spontaneous beat per scan interval of 8 ~ 10 s could be acquired. The data analysis of APs was automated to reduce bias and achieve increased throughput (Supplemental Fig. S1). Pro-arrhythmic risk metrics were defined as depicted in Fig. 1G-I: excitability, stimulation time delay between stimulation pulse and peak AP upstroke (stimulation delay time, stimΔ), rise time of AP upstroke, AP duration (APD) to 30%, 50%, and 80% repolarization (APD_30_, APD_50_, APD_80_), AP duration to end of maximum repolarization rate (APD_MxR_, determined with d^2^F/dt^2^_max_), APD triangulation (APD_tri_ = APD_MxR_ − APD_50_), and presence of EADs (% of microtissues showing EAD).

**Figure 2:**
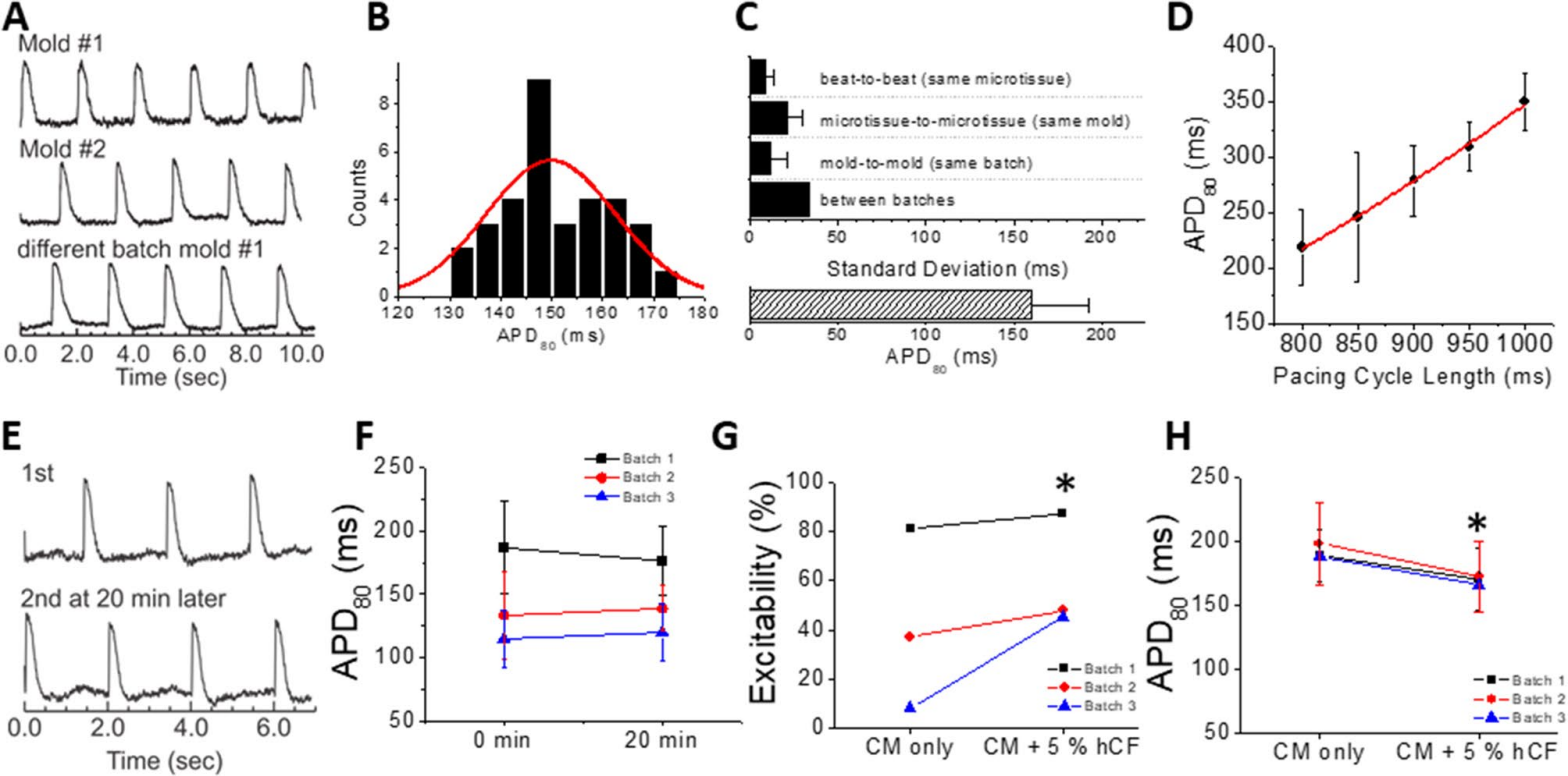
APD variation and stability from hiPSC-CM microtissues in the absence and presence of 5% hCFs. HiPSC-CMs (without lactate purification) were seeded in microwells without or with 5% hCFs. After 6–8 days in 3D culture, microtissues were loaded with di-4-ANEPPS for optical mapping of membrane potentials (V_m_) under paced conditions. (**A**) Representative V_m_ traces from individual microtissues paced at 0.5 Hz in different molds (biological replicates) and different batches (experimental replicates). Values were averaged from all pixels of single microtissues. Only microtissues greater than 60 pixels were analyzed (typically ~ 170 pixels/microtissue). (**B**) APD_80_ distribution from 35 microtissues in a single mold is normally distributed. (**C**) APD variation was assessed for technical, biological, and experimental replicates for APDs measured at 0.5 Hz. The standard deviation from beat-to-beat in the same microtissue (technical replicates) was 8.7 ± 4.8 ms (n > 70 microtissues per batch, n = 7 batches), from microtissue-to-microtissue in the same mold (biological replicates) was 20.9 ± 8.4 (n = 2–3 molds per batch, n = 7 batches), from mold-to-mold in the same batch (also biological variability) was 11.7 ± 8.9 ms (notably less than microtissue-to-microtissue variability; n = 3 molds per batch, n = 7 batches), and from batch-to-batch of differentiated hiPSC-CMs (experimental variability and another level of biological variability) was 33.1 ms (n = 7 batches). Note that the standard deviation reported for batch-to-batch variability does not itself have variation (and therefore no error bar is appropriate). (**D**) The APD restitution curve displays typical rate-dependent increases in APD_80_ with increasing pacing cycle length (800 ms = 1.25 Hz = 75 bpm and 1000 ms = 1 Hz = 60 bpm). (**E**) Representative V_m_ traces recorded from the same microtissue at 2 time points 20 min apart show consistency of AP during optical mapping. (**F**) Quantification of the stability of APD_80_ over 20 min for 3 batches of microtissues (batch #1: 185.2 ± 41.6 vs. 184.0 ± 29.3, *p* = 0.84; batch #2: 114.7 ± 22.2 vs. 176.4 ± 27.1, *p* = 0.46; batch #3: 138.6 ± 17.8 vs. 119.9 ± 22.7, *p* = 0.36). (G, H) Comparison of excitability (**G**) and APD_80_ (**H**) from microtissues within the same batch of hiPSC-CMs without and with 5% hCF (n = 3 hiPSC-CM batches). Values are means ± SD. *P < 0.05 (G, Fisher exact test; H, paired t-test).

Biological variation was low across biological replicates and experimental time (Fig. 2). Example traces show reproducibility (Fig. 2A), and an example of APD distribution across different microtissues from a single mold was normally distributed (Fig. 2B). The Kolmogorov–Smirnov test for 3 molds with a total of 105 cardiac microtissues indicated normal APD distribution (*p* = 0.92). In order to determine the statistical power of this 3D in vitro methodology, we examined the standard deviation as the variability in APD. In microtissues formed with hiPSC-CMs that did not undergo lactate purification (70.3 ± 3.1% cardiac troponin T positive for n = 4 batches), the mean APD_80_ was 160.0 ± 33.1 ms (Fig. 2C, bottom). The standard deviation from batch to batch (“between batches”) was the largest of our biological variability metrics at > 30 ms. Microtissue-to-microtissue and mold-to-mold variability were smaller, and variability in technical replicates beat-to-beat (same microtissue) was < 10 ms (Fig. 2C). Action potential duration showed the expected physiological rate dependence at physiological heart rates (60–75 bpm; Fig. 2D). Power analysis of sample size suggested that n = 17 cardiac microtissues are required to detect a 10% change in APD with 95% confidence, suggesting that testing within a single mold is sufficient for evaluating small but meaningful changes in APD measured in vitro^58^. The sample size can be further reduced by using paired testing (n = 8 based on beat-to-beat variation) on the same mold before and after toxicant exposure. In order to screen cardiotoxicity from the same microtissues before and after toxicant exposure, APDs should be stable between successive recordings. We chose a 20 min incubation period to ensure that the test compound had ample time to diffuse into the 3D cardiac microtissue^59^, which is the limiting factor since typical ion channel blockers bind and act relatively quickly, in less than a minute (for example, E4031 has a binding time constant of 0.8 sec^60^.). Figure 2E,F shows comparable traces and group data for APD_80_ in our model over 20 min, which enabled testing of several doses of toxicant with this acute exposure protocol on the same mold to perform paired t-tests. The addition of 5% hCF to microtissues upon formation improved not only the consistency of formation and clean edges of microtissues (data not shown), but also significantly increased the number of excitable microtissues by more than 10% (Fig. 2G), while slightly shortening APD by 12% (Fig. 2H).

**Figure 3:**
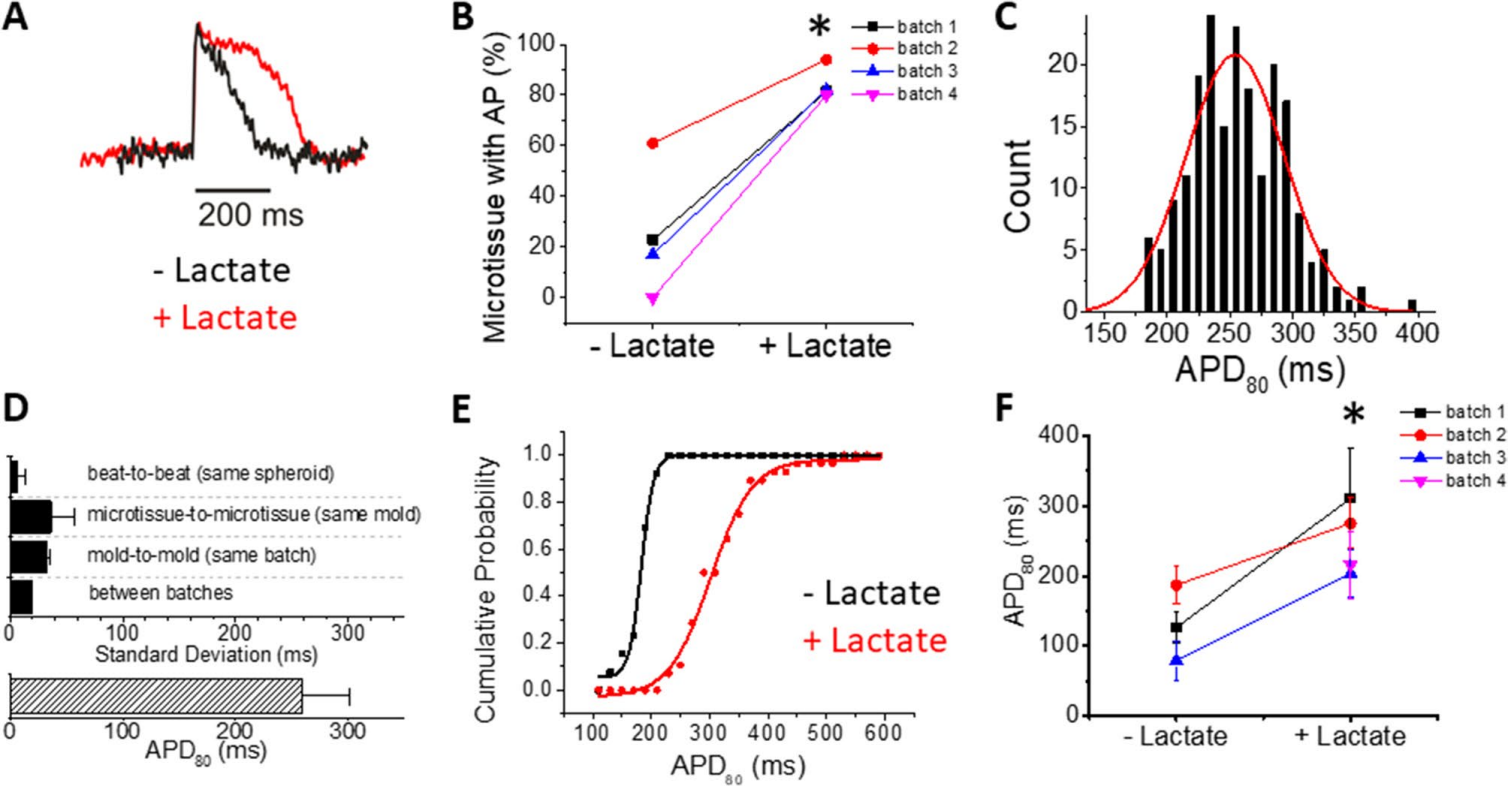
Lactate purification of hiPSC-CMs prior to microtissue formation with 5% hCFs improves excitability and lengthens APD. (**A**) Representative V_m_ traces from microtissues formed from hiPSC-CMs with (red) and without lactate purification (black) plus 5% hCFs. HiPSC-CMs from the same differentiation batch but grown in 2D culture without the lactate selection protocol for 28 days (to match the age of the cells) were used as the most appropriate control for comparison. (**B**) Percent of hiPSC-CM microtissues showing APs during 0.5 Hz pacing increases with lactate purification of hiPSC-CMs (n = 4 batches, minimum 2 molds per batch). (**C**) APD_80_ distribution from 35 microtissues in a single mold with hiPSC-CM_LP_ and 5% hCF. (**D**) The average APD_80_ was 259.0 ± 42.0 ms (n = 201). APD_80_ variation between batches is small with hiPSC-CM_LP_: APDs were measured at 0.5 Hz cycle length pacing. The standard deviation from beat-to-beat in the same microtissue (technical replicates) was 6.4 ± 6.5 ms (n > 35 microtissues per batch, n = 4 batches), from microtissue-to-microtissue in the same mold (biological replicates) was 36.1 ± 21.4 ms (n = 2–3 molds per batch, n = 4 batches), from mold-to-mold in the same batch (also biological variability) was 31.8 ± 3.8 ms (n = 2–3 molds per batch, n = 4 batches), and from batch-to-batch, the standard deviation (another level of biological variability) was 19.3 ms (n = 4 batches). (**E**) Cumulative probability plot for APD_80_ (n = 3–4 batches, at least 2 molds per batch) shows consistently longer APD_80_ with hiPSC-CM_LP_ (red) vs. hiPSC-CM (black). (**F**) Comparison of APDs from microtissues from the same experimental batch (each color) with and without lactate purification (n = 4 batches, ≥ 2 molds per batch). Values are means ± SD. * *P* < 0.05). Note that in batch 4, the microtissues generated from hiPSC-CM without lactate purification were not excitable.

Our model was predictive of expected arrhythmia risk in pilot experiments with known drugs such as I_Kr_ blocker E4031 (data not shown), yet the APD in these microtissues was under 200 ms, and while within the range reported for hPSC-CMs^61^. this was shorter than APD typically recorded in human^62,63^. To improve the reliability and robustness of our arrhythmia model, we selected for and matured our hiPSC-CMs with metabolic-based lactate purification of cardiomyocytes (hiPSC-CM_LP_). Lactate purification lengthened APD (Fig. 3A) and improved the excitability of microtissues when directly compared to experimental controls that were age-matched but not lactate purified (84.4 ± 6.5% for hiPSC-CM_LP_ vs. 25.2 ± 25.8% for hiPSC-CM) (Fig. 3B). Similar to hiPSC-CM microtissues with hCFs, the hiPSC-CM_LP_ microtissues with hCFs have a normal distribution (Kolmogorov–Smirnov test *p* = 0.580; Fig. 3C) and low variability (Fig. 3D). Batch- to-batch standard deviation was reduced by 42% compared to hiPSC-CM microtissues and beat-to-beat APD variation from the same microtissue (technical replicates) was also smaller, whereas microtissue-to-microtissue variability was similar, and mold-to-mold variability was slightly higher than variation in hiPSC-CM tissues (Fig. 3D vs. Figure 2C). The cumulative probability plot for APD_80_ shifted to the right with lactate purification (Fig. 3E), and APD was doubled from 130.6 ± 54 ms in hiPSC-CM microtissues to 263.1 ± 54.9 ms with lactate purification (Fig. 3F). All subsequent experiments were performed with microtissues generated from hiPSC-CMLP and 5% hCFs.

### Model qualification: APD and arrhythmia test with known high-risk and low-risk potassium channel (I_Kr_) blockers

**Figure 4:**
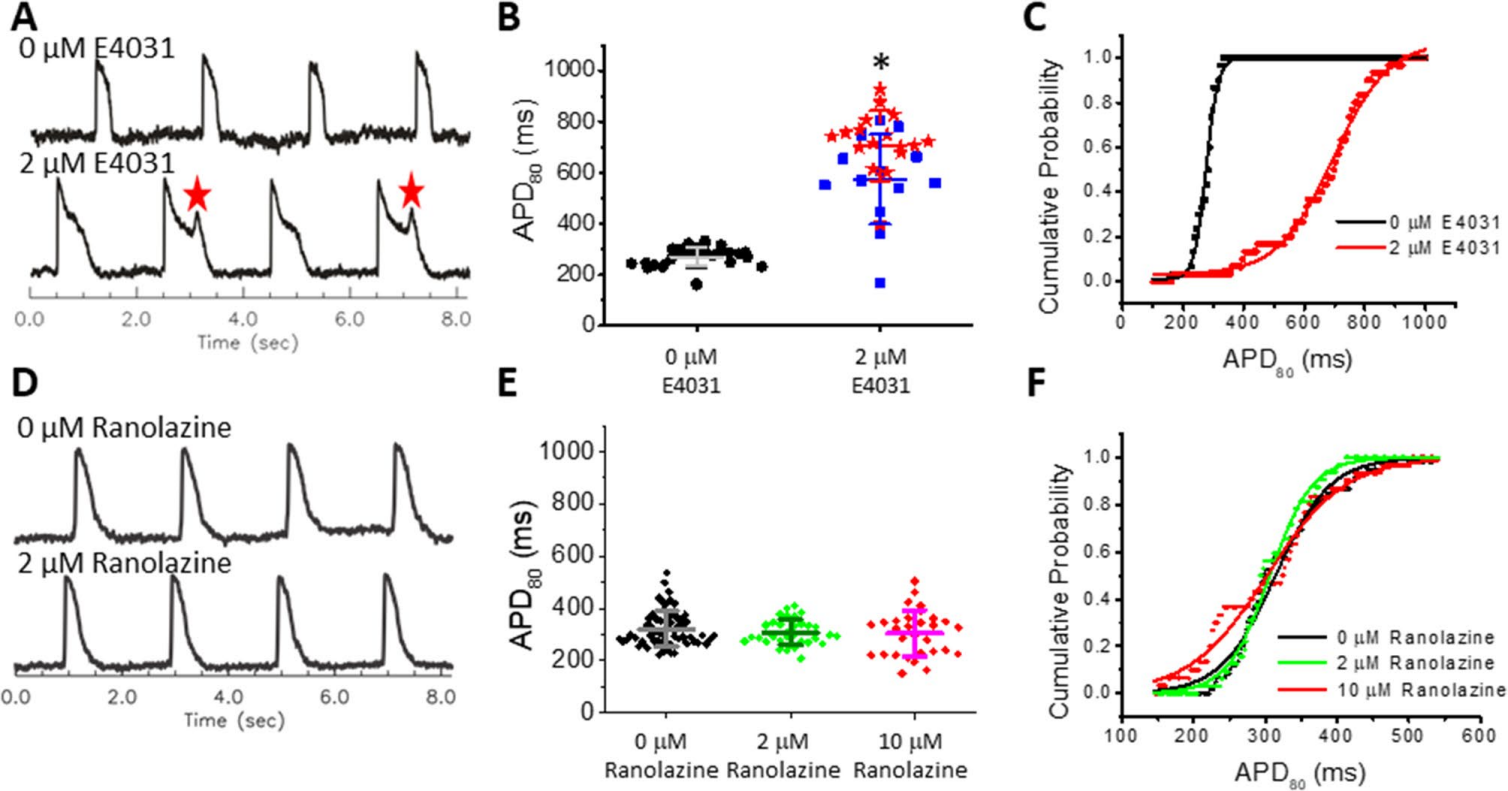
Differentiation between compounds with high risk (E4301) and low risk (ranolazine) for arrhythmia in hiPSC-CM_LP_ with 5% hCF microtissues. (**A**) Representative V_m_ traces from a microtissue (exposed to DMSO vehicle) before and after exposure to E4031 (0 µM and 2 µM, respectively). EADs are marked with red stars. (**B**,**C**) Scatter plot (**B**) and cumulative probability (**C**) of APD_80_ (n = 33 microtissues per group). Values shown by lines in (B) are means ± SD, and APs with EADs are displayed with red stars. 0 μM control was significantly different from each group (vs. no EAD-blue and vs. EAD-red, *p < 0.05). (**D**) Representative V_m_ traces from a microtissue before and after exposure to a therapeutic dose (2 μM) of ranolazine. (**E**,**F**) Scatter plot (**E**) and cumulative probability plot (**F**) for APD_80_ (n = 31). Values shown by lines in (E) are means ± SD. APD was not significantly different between any doses of ranolazine.

To validate the capability of our model to predict arrhythmia risk, we exposed microtissues to the pro-arrhythmic hERG channel blocker E4031. APD_80_ was prolonged under 2 µM E4031 (Fig. 4A,B, Movies S3, S4). Cumulative distribution plots show a rightward shift with E4031 treatment (Fig. 4C). When analyzing single batches of microtissues (n = 35), we confirmed that APD_80_ had a normal distribution of values with Q-Q plots (Supplemental Fig. 2). EADs were present in 54% of microtissues with 2 μM E4031 exposure in the absence of isoproterenol (ISO; Fig. 4A,B, Movie S4). The presence of EADs contributed to a dramatic APD increase (275.2 ± 36 ms vs. 653.3 ± 167.3 ms; Fig. 4C). E4031 prolonged APD in a dose-dependent manner, and in some batches, additional beta-adrenergic stimulation using 100 nM ISO was needed to trigger EADs (Supplemental Fig. 3). Importantly, ISO alone shortened APDs and did not evoke EADs without E4031 (Supplemental Fig. 4A). We further tested blockade of the outward potassium current I_to_ (responsible for phase 1 repolarization) with 4-Aminopyridine (4-AP) and stimulation of L-type calcium channels (responsible for maintaining phase 2 plateau) with the agonist BayK8644, and both prolonged APDs (Supplemental Fig. 4B,C). These results support that cardiac microtissues using hiPSC-CMs and hCFs are an effective platform for screening pro-arrhythmic toxicants.

We further investigated how well this human cardiac microtissue model identifies compounds used medically and already determined to be low risk for arrhythmia in clinically-validated doses in patients even when there is a known capacity to block the hERG channel. One such compound is ranolazine, which is a known hERG channel blocker that has therapeutic value as an anti-arrhythmic drug at mean steady-state blood serum concentrations (C_max_) of 6 μM (2600 ng/mL) with 95% confidence limits of 1 and 14 μM (400 and 6100 ng/mL, respectively)^64,65^. In this concentration range, ranolazine had a minimal effect on APD (Fig. 4D-F), which is in stark contrast to the specific hERG (I_Kr_) blocker E4031 (Fig. 4A-C).

**Figure 5:**
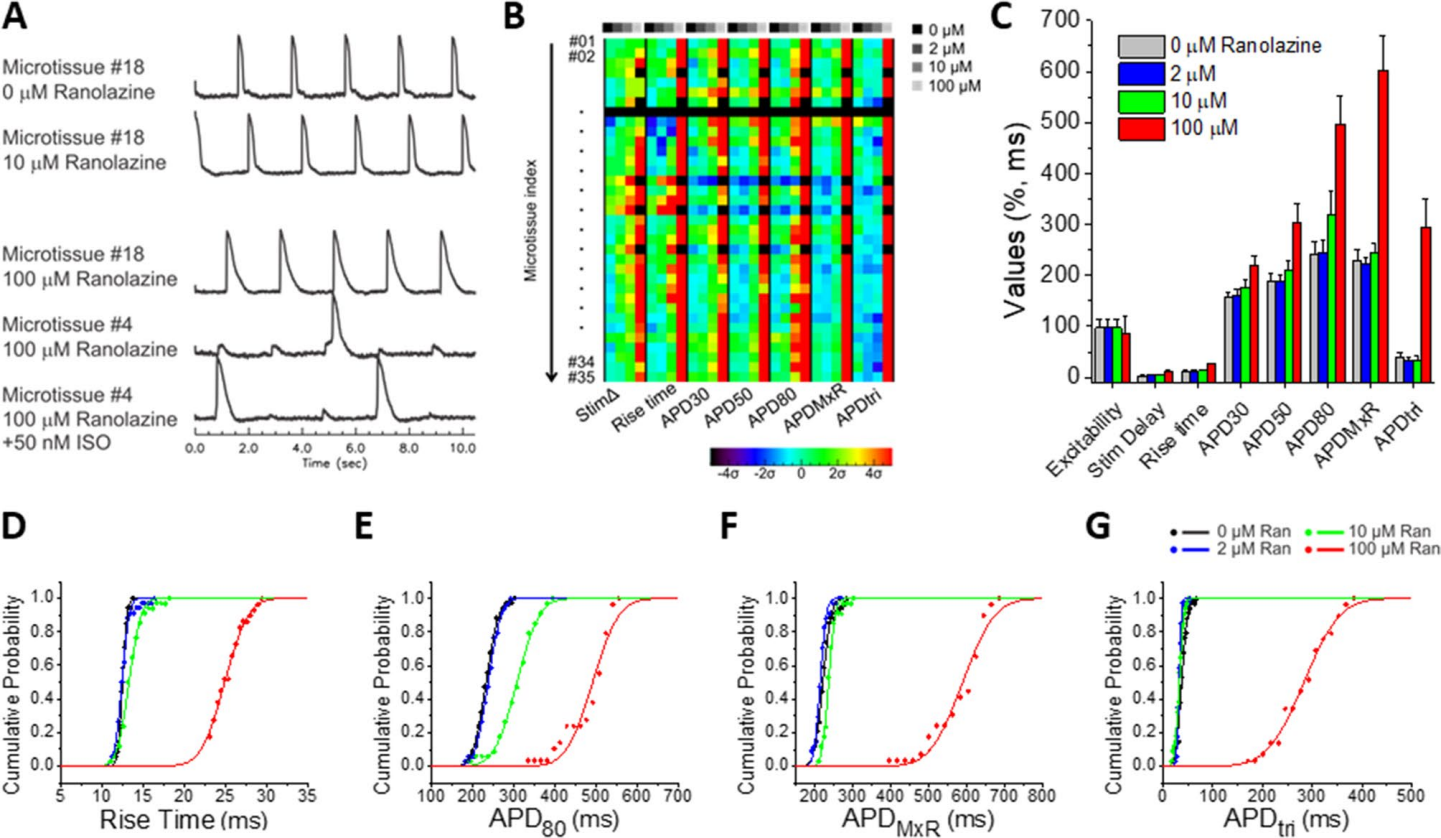
Dose-dependent effects of ranolazine on AP manifested at high concentrations above the therapeutic window in hiPSC-CM_LP_ with 5% hCF microtissues. (**A**) Representative V_m_ traces from 0, 10, 100 μM ranolazine, and 100 μM ranolazine plus 50 nM ISO show AP stability at 10 μM and instability in microtissues exposed to 100 μM Ran with or without ISO . (**B**) AP metric color map shows each microtissue (rows) and seven AP metrics (major columns) with dose-dependent response at indicated concentrations (minor columns). The color displayed shows shifts as measured by the standard deviation (σ) from the average value of the AP metric under the control condition (0 μM). Increases appear in the warm color spectrum (progressing yellow-orange-red) and decreases appear in the cool color spectrum (progressing cyan-blue-purple). Black indicates non-excitable microtissues. When excitability is lost in a microtissue, other metrics could not be measured. (**C**) A summary bar graph for all eight AP metrics shows dose-dependent changes. Values are means ± SD (n = 35). See Supplemental Table 1 (top) for *p* values. (**D**-**G**) Cumulative probability plots for rise time (**D**), APD_80_ (**E**), APD_MxR_ (**F**), and APD_tri_ (**G**) show relative stability up to 10 μM and marked changes and increased variability (shown by the reduced slope of the curve) at 100 μM ranolazine.

Figure 5 shows a more complete assessment of the dose-dependent effects of ranolazine at 0, 2, 10, and 100 μM concentrations on eight AP metrics, and traditional dose response curves are shown in Supplemental Fig. 5A. Figure 5A shows representative examples of AP traces. Figure 5B shows a color map presentation of seven of the AP metrics from 35 microtissues, and group data are shown for each of them plus excitability in Fig. 5C. Ranolazine at therapeutic concentrations (2–10 μM) had statistically significant effects but very small on all AP metrics (Fig. 5C; see Supplemental Table 1 (top) for complete statistical analysis). For example, average APD_MxR_ changes were negligible (− 9 ms and + 15 ms at 2 and 10 μM, respectively) versus control 0 μM (Fig. 5C,F). However, at high concentration (100 μM ranolazine), APD_MXR_ more than doubled (ΔAPD = 357 ms, Fig. 5C,E). Despite prolongation, no EADs were observed with ranolazine even in the presence of ISO (see Fig. 5A bottom trace; 1–2 molds per batch, 2 batches) in contrast to the highly selective hERG channel blocker, E4031 (Fig. 4A,B and Supplemental Fig. 3). 100 μM ranolazine reduced excitability from 97.1% at 0–10 μM to 85.1% at 100 μM, as some of the pacing beats were missed (Fig. 5A-C) and was associated with increased stimulation delay and AP upstroke rise time (Fig. 5B-D) consistent with its therapeutic effects on I_Na_. Statistically significant changes are detected across all metrics and often at all concentrations of ranolazine (Supplemental Table 1), suggesting that this platform is highly sensitive.

### Screening pro-arrhythmic toxicity of environmental toxicant exposure

**Figure 6:**
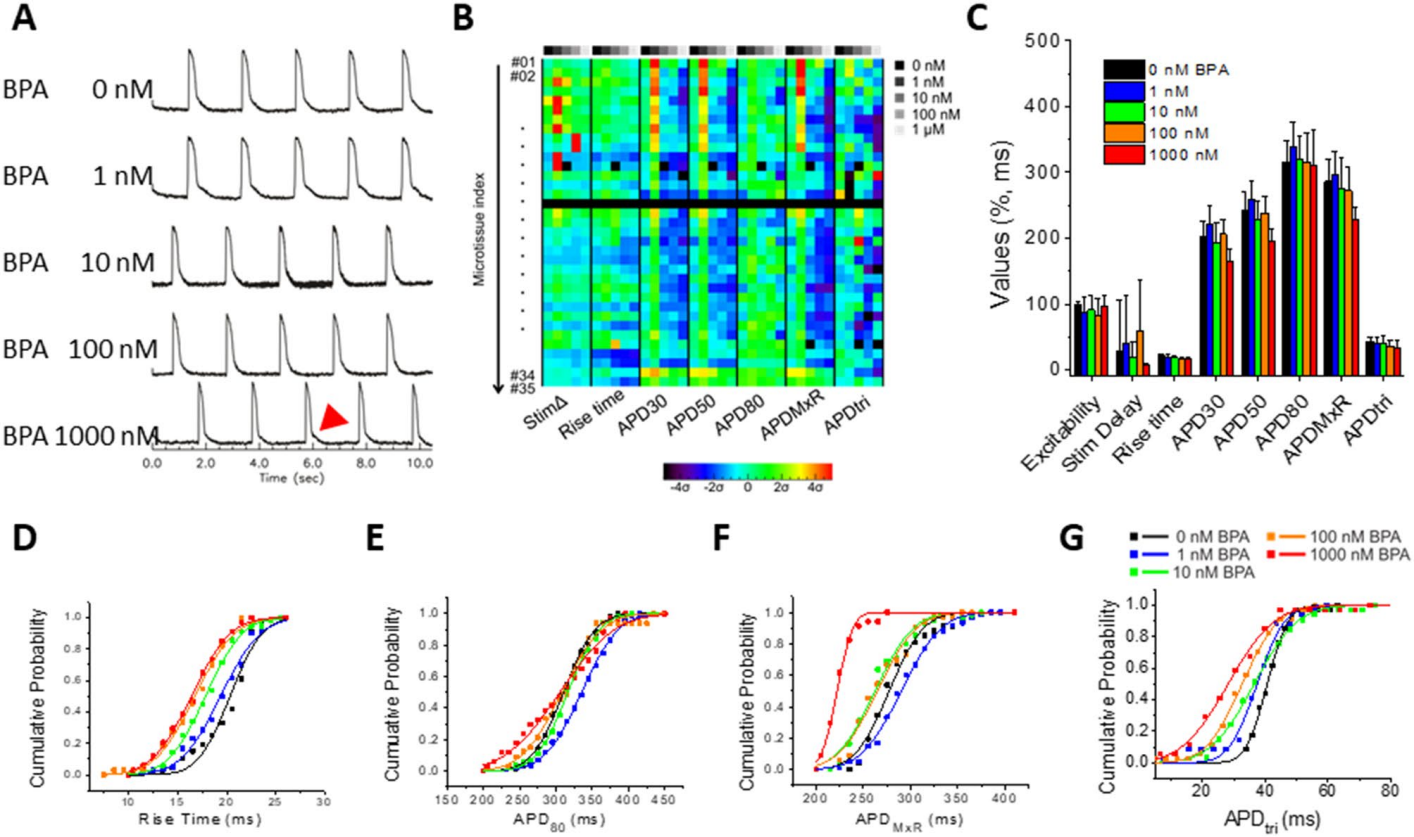
AP changes in response to the environmental chemical BPA in hiPSC-CM_LP_ with 5% hCF microtissues. (**A**) Representative V_m_ traces from 0, 1, 10, 100, and 1000 nM BPA stimulated at 0.5 Hz. (**B**) AP metric color map shows a trend of APD shortening (blue colors) at higher doses and a minority of microtissues having a biphasic effect with increases (red–orange) in stimΔ and APD metrics at 1 nM. Key to the panel is as described in Fig. 5B. (**C**) AP parameter summary bar graph. Values are means ± SD. See Supplemental Table 1 (bottom) for *p* values. (**D**-**G**) Cumulative probability plots for rise time (**D**), APD_80_ (**E**), APD_MxR_ (**F**), and APD_tri_ (**G**).

We investigated whether acute exposure to the environmental pollutant bisphenol A (BPA) alters AP parameters using our human cardiac microtissue platform. BPA has previously been shown to affect the cardiovascular system^66^, but its specific effects on arrhythmia are not well characterized. The response of cardiac microtissues to BPA was complex, altering multiple AP parameters including excitability, stimulation time delay, APDs at different levels of repolarization, APD_MxR_, and APD_tri_ in a dose-dependent manner (Fig. 6). A small but significant increase in APD metrics appeared at the lowest concentration of BPA (1 nM), but BPA gradually shortened APD metrics at higher concentrations (Fig. 6B,C). This biphasic response was most pronounced for APD_MxR_, which increased by 10.5 ms with 1 nM BPA, was similar to no BPA with 10 nM BPA, and decreased by 14.2 ms and 57.5 ms with 100 and 1000 nM BPA (Fig. 6C,F). Similar to APD, a biphasic effect of BPA was observed in a fraction of the microtissues (~ 6 of 35) on stimulation time delay (Fig. 6B, red/orange boxes at 1 nM in StimΔ column), suggesting that complex interactions of BPA on multiple cardiac ion channels involved in both excitation and repolarization was revealed during acute exposure across biological replicates in our model. Interestingly, a divergence between APD_80_ and APD_MxR_ was observed at the highest concentration (1000 nM; Fig. 6E,F) due to the appearance of slowed repolarization near the end of AP repolarization (see 1000 nM trace in Fig. 6A), which indicates that very high BPA exposure may also alter inward rectifier current.

**Figure 7:**
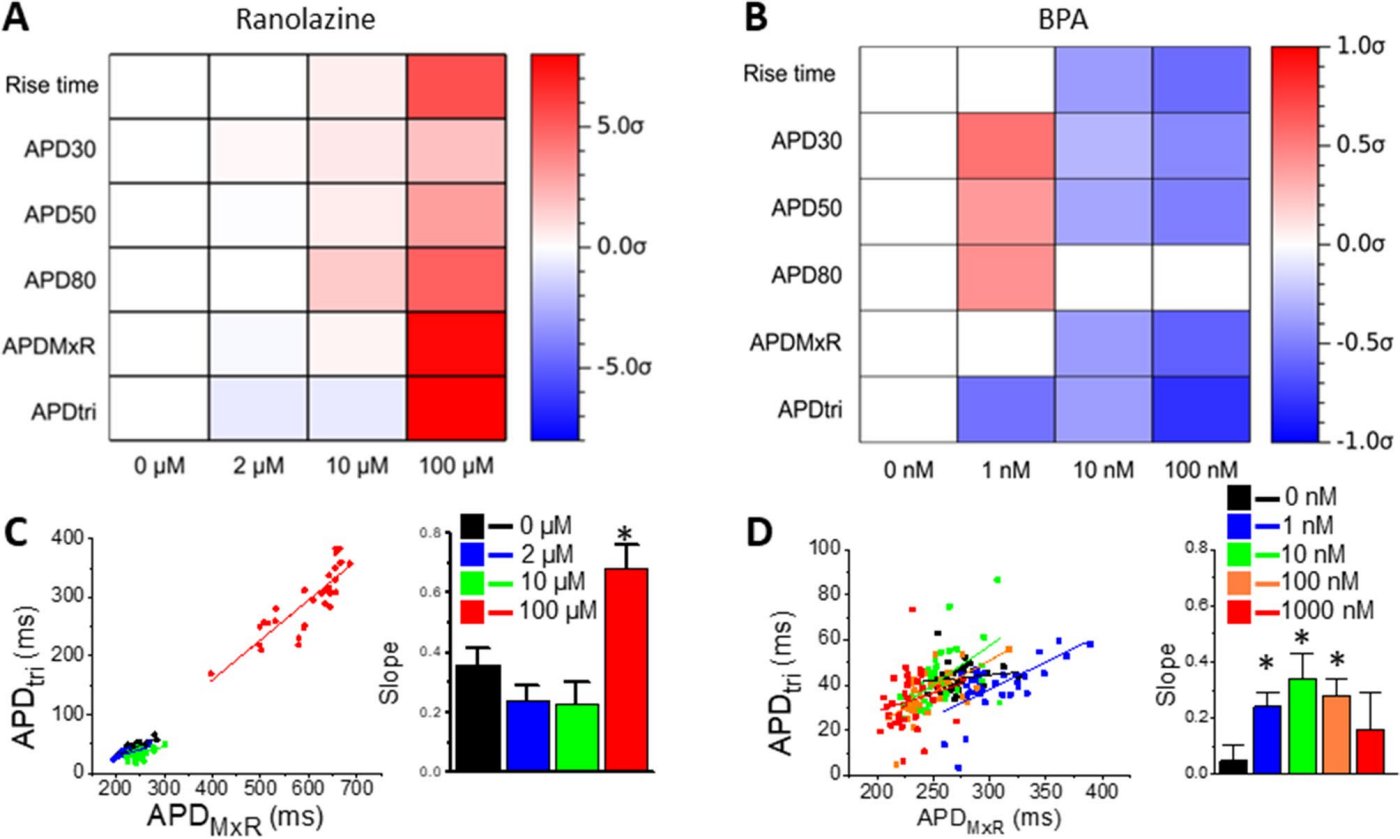
Characteristic AP metric changes induced by Ranolazine and BPA. The color maps of mean difference AP metrics by ranolazine (**A**) and BPA (**B**). The mean differences of AP metrics were normalized by each metric’s standard deviation in control (σ_CTR_). Statistically significant increase and decrease (*p* < 0.05, paired t-test) are presented by red and blue color, respectively. (**C**,**D**) APD_tri_ versus APD_MxR_ plots in response to increasing concentrations of ranolazine (**C**) and BPA (**D**; * *p* < 0.01).

The changes in AP metrics were markedly different between ranolazine and BPA, as reflected in the color maps of AP metric changes in response to each compound (Figs. 5B, 6B, respectively). These drug response signatures are visualized in Fig. 7, where significant changes (red, increased; blue, decreased) and their magnitude (color intensity) of the average AP metrics are shown for each compound at indicated concentrations (see also Supplemental Table 2). Ranolazine has a monophasic increase of rise time and APD_30/50/80_, whereas APD_MxR_ and APD_tri_ are biphasic, suggesting nuanced dose-dependent effects (Fig. 7A,C). These results align well with the known clinical effects at low doses and the drug label warnings of fatal arrhythmias (which appear at high doses). In contrast, BPA shows a biphasic response in APD_30/50/80_ over the concentration range tested (Fig. 7B). Toward understanding the role of hERG channel blockade in these contrasting results, we applied computer simulations to our system. HERG channel blockade has been shown to increase triangulation of AP (APD_tri_) since I_Kr_ peaks during phase 3 repolarization. Our experimental data and computer simulations in Supplemental Fig. 6 show that E4031 (or I_Kr_ blockade in computer modeling) increases the slope of APD_tri_ vs. APD_MxR_ while I_to_ block by 4-AP or augmentation of I_Ca_ by BayK8644 does not alter the slope of APD_tri_ vs. APD_MxR_, supporting that APD_tri_ is an important indicator of hERG channel blockade. Ranolazine increases the slope of APD_tri_ vs. APD_MxR_ associated with significant APD prolongation at 100 µM (Fig. 7C), indicating that hERG blockade becomes significant at this concentration. However, BPA shows biphasic effects on APD_tri_ vs. APD_MxR_, increasing the slope in the range of 1–100 nM but not at 1000 nM. This suggests that BPA potentially blocks hERG starting at very low concentration (1–10 nM) but later alters other ion channels to mask hERG channel blockade and overall these integrated ion channel changes shorten APD. The results presented here support the notion a contention that our 3D human cardiac microtissue model and comprehensive data analysis provide important information regarding cardiotoxicity of unknown compounds.

## Discussion

In this study, we address a critical need in risk assessment of compounds for pro-arrhythmic cardiotoxicity with a 3D human cardiac microtissue model containing purified hiPSC-derived cardiomyocytes and human cardiac fibroblasts. We show robust quantification of eight distinct parameters derived from the action potential waveform and qualify our NAM by confirming physiological responses and differentiation between the high-risk compound E4031 and low-risk drug ranolazine. Importantly, this differentiation enables reduction of false-positive results, as this 3D microtissue model integrates all ion channels and signals in the human cardiomyocytes. Cardiotoxicity can arise from a variety of aberrant signaling so predictive platforms must be suited for situations in which the mechanism of action is not well defined^53^. Testing of a compound with poorly described or unknown cardiac effects such as BPA reveals nuanced responses (Fig. 6), creating a drug response signature, which enables arrhythmic risk and safety evaluation that yields insights into mechanisms of action and is relevant to physiological metrics of health.

Successful pro-arrhythmic drug screening should be able to detect changes in action potential shapes associated with increased triggered activity (EADs and DADs). A major challenge in developing a predictive risk assessment platform is that multiple factors can contribute to initiation of cardiac arrhythmias^67–71^, necessitating that the platform be able to capture multiple pro-arrhythmic changes in AP shape. Here, we report the ability to detect altered excitability (% of captured beats), AP initiation and upstroke, beat-to-beat APD stability, and triangulation of AP shape as well as more standard metrics of prolongation of APD and EAD activity that indicate very high risk for arrhythmia. Our platform goes beyond what is reported by other platforms (such as spontaneous beat rate) that focus on cellular toxicity using live/dead, mitochondrial, and ER imaging^38^, RNAseq for gene expression^72^, or contractile amplitude and kinetics from microtissue edge detection^73^. rather than arrhythmia. Due to our high temporal resolution and signal-to-noise ratio, just a single voltage recording without signal averaging can be used to analyze APD metrics and even detect beat-to-beat instability in APD. These parameters can be compared on the same microtissue before and after compound exposure (20 min) using a paired t-test, which increases statistical power and reduces the number of experiments. Other platforms report the calcium transient, using it as a surrogate metric for the voltage signal, but unlike our approach, this surrogate does not provide mechanistic clues underlying arrhythmia induction. The AP metrics defined in this study are more numerous and less variable than those reported from 2D monolayer assays (using voltage/calcium dyes or microelectrode arrays)^47,49,50^, 3D spheroid assays^45^, or engineered heart tissues (EHTs)^43,44^. These metrics provide integrated data on how the compound impacts electrical activation through changing the AP or Ca handling (Supplemental Table 3). The metrics in our data analysis were selected to account for potential toxicity effects on major ion channels that form cardiac action potentials including sodium channels responsible for AP upstroke (stim delay and rise time), transient outward potassium channels responsible for phase 1 repolarization (APD_30_), L-type Ca channels responsible for maintaining AP plateau and overall duration (APD_30_, APD_50_, APD_80_), delayed-rectifier potassium currents responsible for phase 3 repolarization (APD_MxR_, APD_tri_), and inward rectifier underlying final repolarization (APD_MxR_, APD_80_) and maintaining resting membrane potential that determines excitability together with sodium channels (excitability).

The 3D microenvironment of our microtissues has several advantages to provide quantitative data for predicting pro-arrhythmic toxicity of chemical compounds. The AP recordings here are the averaged behavior from 12,000–24,000 cells that can mask individual cell-to-cell variation to increase robustness of the response. Triggered activity is thought to be difficult to initiate in the 3D environment due to electrotonic dissipation of depolarizing currents during EADs, known as ‘source-sink mismatch’^74^. Therefore, in order to initiate triggered activity, a large number of cells should independently elicit EADs/DADs simultaneously. Our 3D microtissues have a sufficient number of CMs and do generate EADs (Fig. 4 and Supplemental Fig. 3), suggesting that this model replicates the true risks of EAD formation with its multi-cellular 3D environment as is present at the organ level in the human heart. In a direct comparison of the impact of microenvironment on the predictive capacity of an in vitro model using hESC-CMs, Archer et al. showed with a ROC analysis of drug responses to structural cardiotoxins that a 3D microtissue platform has increased specificity versus the same measurements made in 2D monolayers^38^.

Our 3D human cardiotoxicity model has several advantages that enable customization and robust pro-arrhythmic risk evaluation. These advantages are: (1) experimentally determined cell ratios and cell phenotypes (Fig. 1B); (2) self-assembly with organotypic heterocellular interspersion (Fig. 1D,E) while eliminating unnatural substrate or matrix stiffness; (3) robust 3D electrical coupling; (4) rapid optical measurements to track AP shape for highly accurate quantitative metric extraction (Fig. 1F-H); (5) in addition to spontaneous beats, paced action potentials can be analyzed to increase predictability of APD changes under drug; and (6) generation and functional analysis of a large number of individual but consistent microtissues to provide greater throughput and high statistical power in analyses (Figs. 2C, 3D). We have carefully considered the biology of the cardiac cells and employed culturing techniques like moderately longer culture periods and the broadly adopted methods for metabolic selection to purify and mature the hiPSC-CMs^75^, which demonstrate a more mature ventricular physiology in their electrophysiology compared to unpurified populations. While more mature functional responses are observed in our study, future work will require analysis of sarcomeric protein and ion channel expression to characterize this maturation. Further, there is evidence that the presence of non-myocytes from hiPSCs or other sources promote more mature electromechanical function^76^. Incorporation of human cardiac fibroblasts (hCFs) enables heterotypic cell–cell interactions characteristic of the intact myocardium^36,77,78^. While 5% primary adult normal hCF content is used for stable, healthy cardiac electromechanical function based on our previous^79^. and current work, the platform allows for alterations in cell composition (such as other cell types, including endothelial cells) and ratios to mimic physiological and pathophysiological conditions that may be important for toxicity evaluation. While our platform can produce hundreds of microtissues in standard cell culture plates, the low variability beat-to-beat, microtissue-to-microtissue, mold-to-mold, and notably hiPSC-CM differentiation batch-to-batch (Figs. 2C, 3D), that has not been reported in other established models, reduces the required sample size to tens of microtissues per condition, effectively increasing experimental throughput.

Screening of compounds with diverse and known mechanisms of action is widely accepted as critical practice for assessing the validity of in vitro models, but developing metrics to understand biological mechanisms of action is necessary to move towards screening compounds with unknown mechanisms of action^53^. Many established cardiac spheroids and elongated microtissue models have not tried to distinguish responses to high-risk and low-risk drugs^34,45^. Irregular beating patterns in response E-4031 has been shown, but from tissue contraction, not voltage changes to show EADs^42–44^. Some established MEA models have been able to differentiate between the high-risk compound E-4031 and the low-risk drug ranolazine, and our results align well with previous MEA studies that have shown prolonged APD and prevalent EADs with E-4031^80,81^. Ranolazine is consistently shown to modestly increase APD, especially with increasing concentrations, but the presence of EADs is not consistently seen and may be dependent on the hiPSC cell line utilized and the commercial source of MEA platform^32,82^. Computational modeling also predicts increasing APD with ranolazine and does not suggest EADs^83^, and preclinical and clinical data suggest that ranolazine suppresses EADs and DADs^65^. While prolonged APs and EADs are important metrics in arrhythmia risk assessment, previous work in a 2D MEA platform of hiPSC-CMs alone has shown an inability to distinguish compounds that elicit TdP from those that are benign^84^. Because EADs at the cellular scale can be dampened at the tissue scale due to source-sink mismatch with coupled myocytes that are not prone to EADs^85^, 3D platforms may be more predictive of arrhythmia risk. In the clinic, ranolazine rarely causes TdP and is frequently used as anti-arrhythmic treatment despite label warnings of QT prolongation^57,64,86^, suggesting that cardiotoxicity screening must differentiate between high risk compounds and low risk compounds (that could pass safety standards and provide clinical benefit) as we show for E4031 and ranolazine, respectively (Fig. 4). Our platform effectively captures expected responses to standard metrics like APD, yet is less prone to EAD (for example, with ranolazine), suggesting that our 3D microtissue platform and expanded metrics may capture more nuanced responses to more accurately assess risks, predict acceptable exposure levels, and identify multiple modes of toxicity. With ranolazine, we show nuanced dose-dependent effects on the AP quantified by our eight metrics (Fig. 5). This altered excitation reflects ranolazine’s blockage of multiple ion currents including late I_Na_, I_Ca_, I_Na-Ca_, I_Ks_, and I_Kr_^56,87^. To clearly and quantitatively evaluate arrhythmic risk, we further analyzed dose-dependent changes in triangulation of the AP, APD_tri_ (defined by APD_MxR_ – APD_50_), which has been implicated as a pro-arrhythmic predictor^88–90^. Previous studies indicate that an increase in APD_tri_ may originate from blockade of either I_Kr_ or I_Ks_, resulting in delayed phase 3 repolarization^88,91^. Our data also showed that specific I_Kr_ blockade by E4031 increases APD_tri_ but that I_to_ blockade by 4-AP or I_Ca_ activation by BayK8644 had no effect on APD_tri_, which we confirm with computer modeling (Supplemental Fig. 6). The trend of increasing APD_tri_ with a similarly increasing APD would suggest risky I_Kr_ block and can be visualized by plotting APD_tri_ versus APD_MxR_. Previous computer modeling studies predicted that I_Kr_ blockade increases APD_tri_^92,93^. I_Kr_ has unique activation and inactivation kinetics that cause a large rush of outward current, which in turn causes rapid repolarization during phase 3 repolarization. Blocking I_Kr_, therefore, delays phase 3 repolarization and increases APD_tri_. In our experiments, we used APD_tri_ versus APD_MxR_, which is a normalized APD_tri_, and found that APD_tri_ is not always associated with APD prolongation experimentally (e.g., with 4-AP an I_to_ blocker or BayK an I_Ca_ activator). I_Kr_ block (by E4031) specifically increases APD_tri_ in line with predictions by computer simulation studies. The slope of the regression line thus increases when I_Kr_ is blocked and is a clear indicator for dose-dependent arrhythmic toxicity. At lower concentration, ranolazine did not affect slope but at 100 μM it significantly increased (Fig. 7C, bar plot), suggesting that ranolazine blocks I_Kr_, which is in agreement with previous voltage clamp studies that found IC_50_ is 11.5 μM for I_Kr_^87^. However, the less well-known compound BPA showed a biphasic effect on slope of the APD_tri_ vs. APD_MxR_ relationship, peaking at 10 nM and decreasing at higher concentrations, while APD_MxR_ decreases (Fig. 7D). These data suggest that I_Kr_ may be affected at BPA concentrations starting at very low concentration (10 nM) and yet other ion channels and currents may compensate to counteract this effect at higher concentrations. Ultimately, this integrated AP response in human 3D cardiac microtissues with hiPSC-CMs is a defining feature of our model and a significant advantage to be able to predict pro-arrhythmic cardiotoxicity with high sensitivity that will be useful for targeted pharmaceutical development earlier in the drug development pipeline or predictive IVIVE to define safe chemical exposure levels.

It was our aim with this study and in our human cardiotoxicity tissue-engineered model to provide a fit-for-purpose NAM for advancing toxicity risk assessment for pharmaceutical development and safety evaluation of chemicals and environmental toxicants while reducing use of animals, improving mechanistic understanding of cardiotoxicity, and facilitating broad adoption of this simple human in vitro platform. Our platform showed expected changes in arrhythmia metrics to known toxicants and revealed alterations in response to the endocrine disrupting chemical BPA with high sensitivity that suggest cardiac risk and point toward disrupted ion channels as mechanisms of action that may not have been detected in other platforms with more surrogate metrics. Further, automated image analysis and computational approaches including machine learning that have mainly been applied to 2D systems and focus on different outcome metrics such as Ca transient and contractile motion tracking^94–96^. can be applied for AP analyses in our platform to increase its predictive power. Cardiotoxicity risk likely depends on multiple factors including sex, race, and disease status^97^, and toxic effects may be observed to a lesser degree in healthy human subjects compared to patients with comorbidities, defined as ‘hidden toxicity’^18^. These diverse risk factors can be incorporated into our 3D microtissue risk assessment platform through the use of different hiPSC lines; thus exciting opportunities exist to better understand many fundamental mechanisms of toxicity. The predictive value of this platform has the potential to accelerate broad adoption and advances in the regulatory landscape for cardiotoxicity evaluation.

## Methods

### Cardiomyocyte differentiation

Cardiomyocytes (CMs) were differentiated from human induced pluripotent stem cells (hiPSCs; Gibco human female episomal iPSCs) in high-density monolayer cultures using CDM3 medium^98^. and Wnt signal activation and inhibition^99^. (Fig. 1A). Briefly, hiPSCs were treated with 6 µM Chiron 99,021 (Tocris), a glycogen synthase kinase 3 (GSK3) inhibitor at day 1, followed by 5 µM IWP2 (Tocris), a chemical Wnt inhibitor at day 3. Cardiac phenotype, expressed by beating cells, was visible between days 8 and 12 and upon beating, cardiomyocytes were cultured in RPMI 1640 medium with B27 supplement (RPMI + B27; Gibco). Cardiomyocytes differentiated from hiPSCs were used for the production of microtissues between days 14 and 18 of differentiation or were further purified with a lactate protocol. Cardiomyocytes designated for lactate purification were harvested and replated to new culture vessels coated with Matrigel (Corning) in RPMI + B27. These cells were deprived of media change for 4 days and were fed with lactate media (DMEM without glucose, L-glutamine, phenol red, sodium pyruvate and sodium bicarbonate (D-5030, Sigma) + 4 mM L-glutamine, 1 × Non-Essential Amino Acids, 1 × Glutamax and 4 mM lactate, pH 7.4). Lactate purified cells were fed with RPMI + B27 + 1% penicillin/streptomycin (P/S). Cardiac purity was measured by flow cytometry analysis as previously described^100^.

### Human cardiac fibroblast culture

Commercially available human ventricular cardiac fibroblasts (hCFs, Sigma) were maintained and passaged in DMEM/F12 supplemented with 10% fetal bovine serum (FBS), 1% penicillin/streptomycin (P/S), and 4 ng/ml basic fibroblast growth factor (Reprocell). hCFs were incorporated into cardiac microtissues at cell passages P2-P4.

### Fabrication of hydrogels and 3D culture

Scaffold-free 3D spherical microtissues were generated using non-adhesive agarose gels with cylindrical microwells with hemispherical bottoms to guide self-assembly (Fig. 1B). Sterilized 2% (wt/vol) agarose was pipetted into molds designed for 24-well plates with 800-μm-diameter rounded pegs (3D Petri Dish, MicroTissues). After being cooled to room temperature (~ 5 min), the agarose gels were separated from the molds and transferred to single wells of 24-well plates. For equilibration, 1 mL RPMI + B27 + 1% P/S medium was added to each well. Hydrogels were equilibrated for at least 1 h at 37 °C in a humidified incubator with 5% CO_2_. Molds were transferred to 6-well plates for electrical stimulation, and hiPSC-CMs with or without additional 5% hCFs in suspension were added to the center of the hydrogel seeding chamber (420–840 K cells/mold in 35 recesses) and allowed to settle into the recesses for 30 min. Medium (RPMI + B27 with 1% P/S and 10% FBS with 5 μM rock inhibitor (Y27632)) was then added to each well (5 mL). Medium was changed to RPMI + B27 with 1% P/S and 10% FBS at 24–48 h, and cells were cultured for 6–8 days with media changes every other day. During the 3D culture period, the self-assembled microtissues were field stimulated with a 1 Hz, 10.0 V, 4.0 ms duration bipolar pulse train with an Ionoptix C-Pace EP. To study hCF interspersion within tissues, hCF were incubated for 30 min in CellTracker Orange working solution and hiPSC-CM were incubated for 30 min in CellTracker Green working solution before seeding in 3D hydrogels. Spheroids were recovered from hydromolds and transferred to glass bottom dishes. Images were taken with an Olympus FV3000 confocal microscope and processed using ImageJ.

### Microtissue size analysis

Stitched 4 × phase-contrast images of whole 24-well microtissue hydrogels were captured with a Nikon TE2000-U and a black and white/color digital camera (MicroVideo Instruments, Avon, MA). NIS Elements software was used for image acquisition and analysis. Image thresholding and particle size analysis was performed to determine the top view cross-sectional area of individual microtissues across each mold.

### 3D tissue sections and immunohistochemistry

Microtissues in 24-well hydrogels were fixed with 4% (vol/vol) paraformaldehyde (Electron Microscopy Sciences, Hatfield, PA) and 8% (wt/vol) sucrose in PBS overnight at room temperature. Molds were then rinsed twice with PBS and fully equilibrated (as indicated by their sinking, usually over 12 h) with 15% and then 30% (wt/vol) sucrose in PBS. Whole agarose gels containing microtissues were removed from sucrose solution, blotted dry, and embedded in Tissue-Tek CRYO-OCT Compound (Sakura). Blocks were stored at − 80 °C, sectioned on a Leica CM3050 cryostat microtome (Leica Biosystems, Buffalo Grove, IL) into 10-μm-thick sections, and placed on Superfrost Plus slides. After being air dried for 15 min, sections were post-fixed in 4% paraformaldehyde in PBS. For immunofluorescent staining at room temperature, frozen sections were rinsed 3 times for 5 min with 1 × PBS wash buffer. Non-specific binding was blocked with 1.5% goat serum for 1 h, followed by 24 h incubation in primary and followed by a 1 h incubation in secondary antibodies diluted in 1.5% goat serum. Primary antibodies were directed against cardiac troponin I (cTnI, 1:100, Abcam ab47003) and vimentin (1:100, Sigma V6630), and secondary antibodies were conjugated to Alexa Fluor 488 or Alexa Fluor 594 (1:200, Invitrogen). Coverslips were mounted with Vectashield mounting medium with DAPI. Images were taken with an Olympus FV3000 Confocal Microscope and processed using ImageJ.

### Optical mapping and automated action potential analysis

Microtissues were loaded with voltage-sensitive di-4-ANEPPS (5 μM for 10 min at 35 °C) for measurements of membrane potential (*V*_m_). Fluorescence images were acquired at 979 frames/s using a Photometrics Evolve + 128 EMCCD camera (2 × 2 binning to 64 × 64 pixels, 18.7 × 18.7-μm^2^ resolution, 1.2 × 1.2-mm^2^ field of view) and an Olympus MXV10 macroview optical system^101^. Typically, four microtissues were recorded simultaneously per scan at this magnification. A step-by-step illustration of automated data analysis is available in Supplemental Fig. 1. Briefly, the pixels with APs were identified from Fast Fourier transformation (FFT) of fluorescence signals. After appropriate thresholding and image segmentation, the region of each microtissue was grouped and the fluorescence signals from the pixels in the same microtissue were average and used for AP analysis (Supplemental Fig. 1).

### Validation and screening of toxicants for arrhythmogenic risk

A single mold of microtissues was mounted on a temperature-controlled chamber (Dual Automatic Temperature Controller TC-344B, Warner Instrument) to maintain 35 ± 1 °C and bathed with Tyrode’s solution containing (in mM) 140 NaCl, 5.1 KCl, 1 MgCl_2_, 1 CaCl_2_, 0.33 NaH_2_PO_4_, 5 HEPES, and 7.5 glucose. Microtissues were stimulated with a platinum field stimulation electrode (Supplemental Fig. 7, Myopacer EP field stimulator, IonOptix, Milton, MA). The test compounds including E4031, 4-AP, BayK8644, ISO were purchased from Sigma Aldrich and dissolved in 100% DMSO to prepare 0.01–0.5 mM stock solutions. BPA was dissolved in 10% ethanol stock solution and diluted in Tyrode solution to the final concentration. Microtissues were exposed to vehicle (DMSO or ethanol) and the indicated concentrations of test compounds for 20 min, and action potentials were measured as described above.

### Statistical analyses

The data from optical mapping are expressed as mean ± SD for n microtissues unless otherwise indicated. Statistical analyses were performed using Student’s two tailed paired and unpaired *t* test. *P* values of 0.05 were considered statistically significant. Normality test was done using Kolmogorov–Smirnov test. The test for the equality of regression coefficients was done using Z statistics of two slopes and SE as described^102,103^.

## Supplementary Information

Supplementary Video 1.

Supplementary Video 2.

Supplementary Video 3.

Supplementary Video 4.

Supplementary information.

## References

[1] Abbott GW, Roepke TK (2008). Pharmacogenetics of drug-induced arrhythmias. Expert. Rev. Clin. Pharmacol..

[2] Barnes BJ, Hollands JM (2010). Drug-induced arrhythmias. Crit. Care Med..

[3] Bossu A, van der Heyden MA, de Boer TP, Vos MA (2016). A 2015 focus on preventing drug-induced arrhythmias. Expert. Rev. Cardiovasc. Ther..

[4] Fenichel RR (2004). Drug-induced torsades de pointes and implications for drug development. J. Cardiovasc. Electrophysiol..

[5] Lee A, Pickham D (2016). Basic cardiac electrophysiology and common drug-induced arrhythmias. Crit. Care Nurs. Clin. N. Am.

[6] Recanatini M, Poluzzi E, Masetti M, Cavalli A, De Ponti F (2005). QT prolongation through hERG K(+) channel blockade: current knowledge and strategies for the early prediction during drug development. Med. Res. Rev..

[7] Haverkamp W (2000). The potential for QT prolongation and pro-arrhythmia by non-anti-arrhythmic drugs: clinical and regulatory implications. Report on a Policy Conference of the European Society of Cardiology. Cardiovasc. Res..

[8] Prüss-Üstün A, Corvalán C (2006). Preventing Disease Through Healthy Environments: Towards an Estimate of the Environmental Burden of Disease.

[9] Virani SS (2020). Heart disease and stroke statistics-2020 update: A report from the American Heart Association. Circulation.

[10] Tanner MR, Beeton C (2018). Differences in ion channel phenotype and function between humans and animal models. Front. Biosci. (Landmark Ed).

[11] Bracken MB (2009). Why animal studies are often poor predictors of human reactions to exposure. J. R. Soc. Med..

[12] Haraguchi Y, Ohtsuki A, Oka T, Shimizu T (2015). Electrophysiological analysis of mammalian cells expressing hERG using automated 384-well-patch-clamp. BMC Pharmacol. Toxicol..

[13] Wacker S, Noskov SY (2018). Performance of machine learning algorithms for qualitative and quantitative prediction drug blockade of hERG1 channel. Comput. Toxicol..

[14] Alinejad S, Kazemi T, Zamani N, Hoffman RS, Mehrpour O (2015). A systematic review of the cardiotoxicity of methadone. EXCLI J..

[15] Heranval A (2016). Drugs with potential cardiac adverse effects: Retrospective study in a large cohort of parkinsonian patients. Rev. Neurol. (Paris).

[16] Sun C, Brice JA, Clark RF (2018). Brugada-type pattern on electrocardiogram associated with high-dose loperamide abuse. J. Emerg. Med..

[17] Ramalho D, Freitas J (2018). Drug-induced life-threatening arrhythmias and sudden cardiac death: A clinical perspective of long QT, short QT and Brugada syndromes. Rev. Port. Cardiol..

[18] Ferdinandy P (2019). Definition of hidden drug cardiotoxicity: paradigm change in cardiac safety testing and its clinical implications. Eur. Heart J..

[19] Singh BN (2006). Amiodarone: a multifaceted antiarrhythmic drug. Curr. Cardiol. Rep..

[20] Singh BN, Wadhani N (2004). Antiarrhythmic and proarrhythmic properties of QT-prolonging antianginal drugs. J. Cardiovasc. Pharmacol. Ther..

[21] Wu L (2008). Augmentation of late sodium current unmasks the proarrhythmic effects of amiodarone. Cardiovasc. Res..

[22] Kurokawa YK, George SC (2016). Tissue engineering the cardiac microenvironment: Multicellular microphysiological systems for drug screening. Adv. Drug Deliv. Rev..

[23] Strickland J (2018). Status of acute systemic toxicity testing requirements and data uses by U.S. regulatory agencies. Regul. Toxicol. Pharmacol..

[24] Methods, I. C. C. o. t. V. o. A. A Strategic Roadmap for Establishing New Approaches to Evaluate the Safety of Chemicals and Medical Products in the United States. 10.22427/NTP-ICCVAM-ROADMAP2018 (2018).

[25] Parish ST (2020). An evaluation framework for new approach methodologies (NAMs) for human health safety assessment. Regul Toxicol Pharmacol.

[26] Sager PT, Gintant G, Turner JR, Pettit S, Stockbridge N (2014). Rechanneling the cardiac proarrhythmia safety paradigm: a meeting report from the Cardiac Safety Research Consortium. Am. Heart J..

[27] Strauss DG (2019). Comprehensive in vitro proarrhythmia assay (CiPA) Update from a cardiac safety research consortium/health and environmental sciences institute/FDA meeting. Ther. Innov. Regul. Sci..

[28] Gintant G, Sager PT, Stockbridge N (2016). Evolution of strategies to improve preclinical cardiac safety testing. Nat. Rev. Drug Discov..

[29] Chen IY, Matsa E, Wu JC (2016). Induced pluripotent stem cells: at the heart of cardiovascular precision medicine. Nat. Rev. Cardiol..

[30] Gintant G (2019). Use of human induced pluripotent stem cell-derived cardiomyocytes in preclinical cancer drug cardiotoxicity testing: A scientific statement from the American Heart Association. Circ. Res..

[31] Sinnecker D, Laugwitz KL, Moretti A (2014). Induced pluripotent stem cell-derived cardiomyocytes for drug development and toxicity testing. Pharmacol. Ther..

[32] Blinova K (2018). International multisite study of human-induced pluripotent stem cell-derived cardiomyocytes for drug proarrhythmic potential assessment. Cell Rep..

[33] Nguyen DC (2014). Microscale generation of cardiospheres promotes robust enrichment of cardiomyocytes derived from human pluripotent stem cells. Stem Cell Rep..

[34] Beauchamp P (2015). Development and characterization of a scaffold-free 3D spheroid model of induced pluripotent stem cell-derived human cardiomyocytes. Tissue Eng. Part C Methods.

[35] Desroches BR (2012). Functional scaffold-free 3-D cardiac microtissues: a novel model for the investigation of heart cells. Am. J. Physiol. Heart Circ. Physiol..

[36] Kofron CM (2017). Gq-activated fibroblasts induce cardiomyocyte action potential prolongation and automaticity in a three-dimensional microtissue environment. Am. J. Physiol. Heart Circ. Physiol..

[37] Polonchuk L (2017). Cardiac spheroids as promising in vitro models to study the human heart microenvironment. Sci. Rep..

[38] Archer CR (2018). Characterization and validation of a human 3D cardiac microtissue for the assessment of changes in cardiac pathology. Sci. Rep..

[39] Verheijen M (2018). Bringing in vitro analysis closer to in vivo: Studying doxorubicin toxicity and associated mechanisms in 3D human microtissues with PBPK-based dose modelling. Toxicol. Lett..

[40] Ravenscroft SM, Pointon A, Williams AW, Cross MJ, Sidaway JE (2016). Cardiac non-myocyte cells show enhanced pharmacological function suggestive of contractile maturity in stem cell derived cardiomyocyte microtissues. Toxicol. Sci..

[41] Pointon A (2017). From the cover: High-throughput imaging of cardiac microtissues for the assessment of cardiac contraction during drug discovery. Toxicol. Sci..

[42] Zhao, Y. *et al.* A platform for generation of chamber-specific cardiac tissues and disease modeling. *Cell* **176**, 913–927 e918. 10.1016/j.cell.2018.11.042 (2019). 10.1016/j.cell.2018.11.042 PMC6456036 30686581

[43] Feric NT (2019). Engineered cardiac tissues generated in the Biowire II: A platform for human-based drug discovery. Toxicol. Sci..

[44] Takeda M (2018). Development of in vitro drug-induced cardiotoxicity assay by using three-dimensional cardiac tissues derived from human induced pluripotent stem cells. Tissue Eng. Part C Methods.

[45] Bergstrom G, Christoffersson J, Schwanke K, Zweigerdt R, Mandenius CF (2015). Stem cell derived in vivo-like human cardiac bodies in a microfluidic device for toxicity testing by beating frequency imaging. Lab. Chip.

[46] Sirenko O (2017). In vitro cardiotoxicity assessment of environmental chemicals using an organotypic human induced pluripotent stem cell-derived model. Toxicol. Appl. Pharmacol..

[47] Jahnke HG (2013). A novel 3D label-free monitoring system of hES-derived cardiomyocyte clusters: a step forward to in vitro cardiotoxicity testing. PLoS ONE.

[48] Takasuna K (2017). Comprehensive in vitro cardiac safety assessment using human stem cell technology: Overview of CSAHi HEART initiative. J. Pharmacol. Toxicol. Methods.

[49] Fleischer S, Jahnke HG, Fritsche E, Girard M, Robitzki AA (2019). Comprehensive human stem cell differentiation in a 2D and 3D mode to cardiomyocytes for long-term cultivation and multiparametric monitoring on a multimodal microelectrode array setup. Biosens. Bioelectron..

[50] Asahi Y (2019). Electrophysiological evaluation of pentamidine and 17-AAG in human stem cell-derived cardiomyocytes for safety assessment. Eur. J. Pharmacol..

[51] Navarrete EG (2013). Screening drug-induced arrhythmia [corrected] using human induced pluripotent stem cell-derived cardiomyocytes and low-impedance microelectrode arrays. Circulation.

[52] Pistollato F, Bremer-Hoffmann S, Healy L, Young L, Stacey G (2012). Standardization of pluripotent stem cell cultures for toxicity testing. Expert. Opin. Drug Metab. Toxicol..

[53] Magdy T, Schuldt AJT, Wu JC, Bernstein D, Burridge PW (2018). Human induced pluripotent stem cell (hiPSC)-derived cells to assess drug cardiotoxicity: Opportunities and problems. Annu. Rev. Pharmacol. Toxicol..

[54] Berenfeld O, Efimov I (2019). Optical Mapping. Card Electrophysiol. Clin..

[55] Salama G, Choi BR (2007). Imaging ventricular fibrillation. J. Electrocardiol..

[56] Crumb WJ, Vicente J, Johannesen L, Strauss DG (2016). An evaluation of 30 clinical drugs against the comprehensive in vitro proarrhythmia assay (CiPA) proposed ion channel panel. J. Pharmacol. Toxicol. Methods.

[57] Vicente J (2015). Comprehensive T wave morphology assessment in a randomized clinical study of dofetilide, quinidine, ranolazine, and verapamil. J. Am. Heart Assoc..

[58] Nachimuthu S, Assar MD, Schussler JM (2012). Drug-induced QT interval prolongation: mechanisms and clinical management. Ther. Adv. Drug Saf..

[59] Achilli TM, McCalla S, Meyer J, Tripathi A, Morgan JR (2014). Multilayer spheroids to quantify drug uptake and diffusion in 3D. Mol Pharm..

[60] Clay JR, Ogbaghebriel A, Paquette T, Sasyniuk BI, Shrier A (1995). A quantitative description of the E-4031-sensitive repolarization current in rabbit ventricular myocytes. Biophys. J..

[61] Ma J (2011). High purity human-induced pluripotent stem cell-derived cardiomyocytes: electrophysiological properties of action potentials and ionic currents. Am. J. Physiol. Heart Circ. Physiol..

[62] Taggart P, Sutton PM, Boyett MR, Lab M, Swanton H (1996). Human ventricular action potential duration during short and long cycles. Rapid modulation by ischemia. Circulation.

[63] Franz MR, Swerdlow CD, Liem LB, Schaefer J (1988). Cycle length dependence of human action potential duration in vivo. Effects of single extrastimuli, sudden sustained rate acceleration and deceleration, and different steady-state frequencies. J. Clin. Invest..

[64] Gilead Sciences, Inc., Foster City, CA (2015).

[65] Gupta T, Khera S, Kolte D, Aronow WS, Iwai S (2015). Antiarrhythmic properties of ranolazine: A review of the current evidence. Int. J. Cardiol..

[66] Gao X, Wang HS (2014). Impact of bisphenol a on the cardiovascular system - epidemiological and experimental evidence and molecular mechanisms. Int. J. Environ. Res. Public Health.

[67] Elson J, Mason JW (1986). General concepts and mechanisms of ventricular tachycardia. Cardiol. Clin..

[68] Ebinger MW, Krishnan S, Schuger CD (2005). Mechanisms of ventricular arrhythmias in heart failure. Curr. Heart Fail. Rep..

[69] Makati KJ (2008). Advances in mechanisms of atrial fibrillation: structural remodeling, high-frequency fractionated electrograms, and reentrant AF drivers. J. Interv. Card Electrophysiol..

[70] Sattler SM (2019). Ventricular arrhythmias in first acute myocardial infarction: Epidemiology, mechanisms, and interventions in large animal models. Front. Cardiovasc. Med..

[71] Pellman J, Zhang J, Sheikh F (2016). Myocyte-fibroblast communication in cardiac fibrosis and arrhythmias: Mechanisms and model systems. J. Mol. Cell. Cardiol..

[72] Richards DJ (2020). Human cardiac organoids for the modelling of myocardial infarction and drug cardiotoxicity. Nat. Biomed. Eng..

[73] Beauchamp P (2020). 3D Co-culture of hiPSC-derived cardiomyocytes with cardiac fibroblasts improves tissue-like features of cardiac spheroids. Front. Mol. Biosci..

[74] Xie Y, Sato D, Garfinkel A, Qu Z, Weiss JN (2010). So little source, so much sink: requirements for after depolarizations to propagate in tissue. Biophys. J..

[75] Lin B (2017). Culture in glucose-depleted medium supplemented with fatty acid and 3,3',5-triiodo-l-thyronine facilitates purification and maturation of human pluripotent stem cell-derived cardiomyocytes. Front. Endocrinol. (Lausanne).

[76] Tiburcy M (2017). Defined engineered human myocardium with advanced maturation for applications in heart failure modeling and repair. Circulation.

[77] Kofron CM, Mende U (2017). In vitro models of the cardiac microenvironment to study myocyte and non-myocyte crosstalk: bioinspired approaches beyond the polystyrene dish. J. Physiol..

[78] Zuppinger C (2019). 3D cardiac cell culture: A critical review of current technologies and applications. Front. Cardiovasc. Med..

[79] Rupert CE, Kim TY, Choi BR, Coulombe KLK (2020). Human cardiac fibroblast number and activation state modulate electromechanical function of hiPSC-cardiomyocytes in engineered myocardium. Stem Cells Int..

[80] Pfeiffer-Kaushik ER (2019). Electrophysiological characterization of drug response in hSC-derived cardiomyocytes using voltage-sensitive optical platforms. J. Pharmacol. Toxicol. Methods.

[81] Millard D (2018). Cross-site reliability of human induced pluripotent stem cell-derived cardiomyocyte based safety assays using microelectrode arrays: Results from a blinded CiPA pilot study. Toxicol. Sci..

[82] Gilchrist KH, Lewis GF, Gay EA, Sellgren KL, Grego S (2015). High-throughput cardiac safety evaluation and multi-parameter arrhythmia profiling of cardiomyocytes using microelectrode arrays. Toxicol. Appl. Pharmacol..

[83] Sahli Costabal F, Yao J, Kuhl E (2018). Predicting drug-induced arrhythmias by multiscale modeling. Int. J. Numer. Method Biomed. Eng..

[84] Qu Y, Vargas HM (2015). Proarrhythmia risk assessment in human induced pluripotent stem cell-derived cardiomyocytes using the maestro MEA platform. Toxicol. Sci..

[85] Weiss JN, Garfinkel A, Karagueuzian HS, Chen PS, Qu Z (2010). Early afterdepolarizations and cardiac arrhythmias. Heart Rhythm.

[86] Johannesen L (2014). Differentiating drug-induced multichannel block on the electrocardiogram: randomized study of dofetilide, quinidine, ranolazine, and verapamil. Clin. Pharmacol. Ther..

[87] Antzelevitch C (2004). Electrophysiological effects of ranolazine, a novel antianginal agent with antiarrhythmic properties. Circulation.

[88] Guerard NC, Traebert M, Suter W, Dumotier BM (2008). Selective block of IKs plays a significant role in MAP triangulation induced by IKr block in isolated rabbit heart. J. Pharmacol. Toxicol. Methods.

[89] Grunnet M (2010). Repolarization of the cardiac action potential. Does an increase in repolarization capacity constitute a new anti-arrhythmic principle?. Acta Physiol. (Oxf).

[90] Hondeghem LM, Carlsson L, Duker G (2001). Instability and triangulation of the action potential predict serious proarrhythmia, but action potential duration prolongation is antiarrhythmic. Circulation.

[91] Trenor B (2013). In silico assessment of drug safety in human heart applied to late sodium current blockers. Channels.

[92] Romero L, Pueyo E, Fink M, Rodriguez B (2009). Impact of ionic current variability on human ventricular cellular electrophysiology. Am. J. Physiol. Heart Circ. Physiol..

[93] Dutta S (2017). Optimization of an in silico cardiac cell model for proarrhythmia risk assessment. Front. Physiol..

[94] Juhola M, Penttinen K, Joutsijoki H, Aalto-Setala K (2021). Analysis of drug effects on iPSC cardiomyocytes with machine learning. Ann. Biomed. Eng..

[95] McKeithan WL (2017). An automated platform for assessment of congenital and drug-induced arrhythmia with hiPSC-derived cardiomyocytes. Front. Physiol..

[96] Hoang P (2018). Quantitatively characterizing drug-induced arrhythmic contractile motions of human stem cell-derived cardiomyocytes. Biotechnol. Bioeng..

[97] Zeng H, Wang J, Clouse H, Lagrutta A, Sannajust F (2019). Resolving the reversed rate effect of calcium channel blockers on human-induced pluripotent stem cell-derived cardiomyocytes and the impact on in vitro cardiac safety evaluation. Toxicol. Sci..

[98] Burridge PW (2011). A universal system for highly efficient cardiac differentiation of human induced pluripotent stem cells that eliminates interline variability. PLoS ONE.

[99] Lian X (2012). Robust cardiomyocyte differentiation from human pluripotent stem cells via temporal modulation of canonical Wnt signaling. Proc. Natl. Acad. Sci U. S. A..

[100] Rupert CE, Irofuala C, Coulombe KLK (2020). Practical adoption of state-of-the-art hiPSC-cardiomyocyte differentiation techniques. PLoS ONE.

[101] Kim TY (2018). Directed fusion of cardiac spheroids into larger heterocellular microtissues enables investigation of cardiac action potential propagation via cardiac fibroblasts. PLoS ONE.

[102] Cohen J, Cohen P, West SG, Aiken LS (2003). Applied Multiple Regression/Correlation Analysis for the Behavioral Sciences.

[103] Paternoster R, Brame R, Mazerolle P, Piquero AR (1998). Using the correct statistical test for the equality of regression coefficients. Criminology.

