# Supplementary material for: A predictive in vitro risk assessment platform for pro-arrhythmic toxicity using human 3D cardiac microtissues
Source: Sci Rep. 2021 May 13;11:10228. doi: 10.1038/s41598-021-89478-9 (PMC8119415; doi:10.1038/s41598-021-89478-9)
Supplement: Supplementary file 5 — Supplementary information. [file 41598_2021_89478_MOESM5_ESM.docx]

**Supplemental Material**

A predictive in vitro risk assessment platform for pro-arrhythmic toxicity using human 3D cardiac microtissues

Celinda M. Kofron*^1^, Tae Yun Kim*^2^, Fabiola Munarin^1^, Arvin H. Soepriatna^1^, Rajeev J. Kant^1^, Ulrike Mende^2^, Bum-Rak Choi^§2^, Kareen L.K. Coulombe^§1^

^1^Center for Biomedical Engineering, School of Engineering, Brown University, Providence, RI

^2^Cardiovascular Research Center, Cardiovascular Institute, Rhode Island Hospital and Alpert Medical School of Brown University, Providence, RI

* Contributed equally to this study.

^§^ Contributed equally to this study, co-corresponding authors.


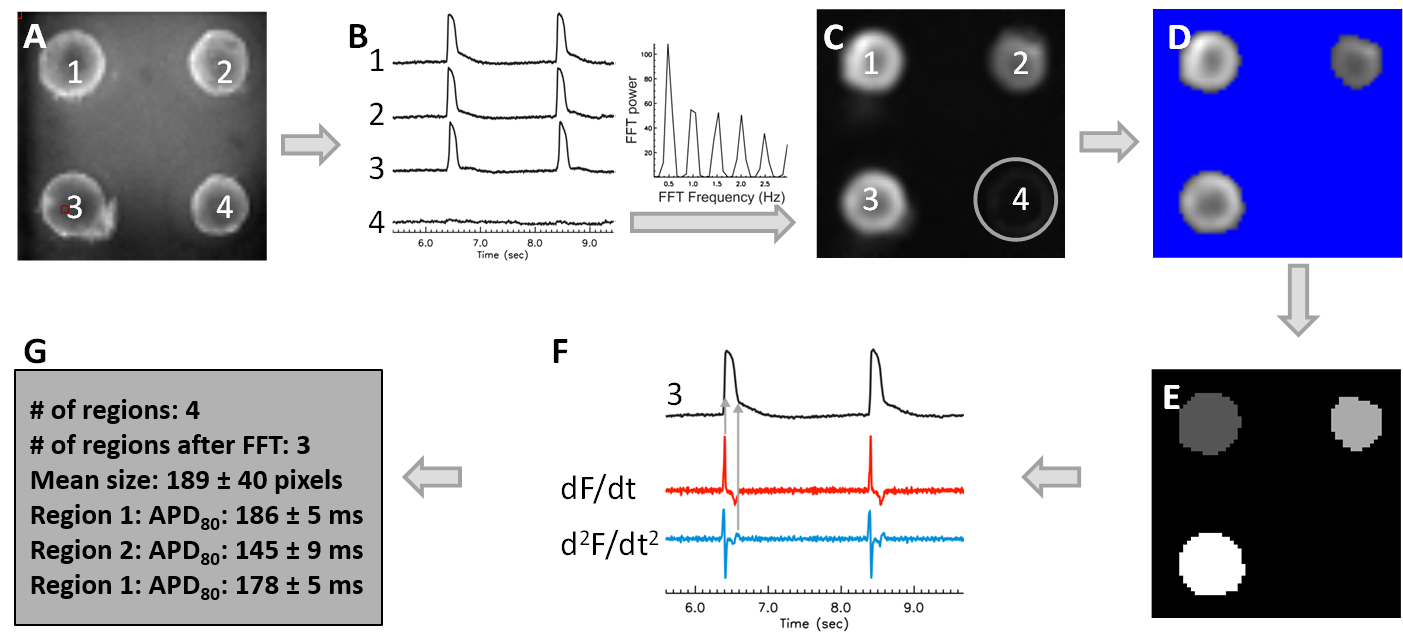


**Supplemental Figure 1. Automated analysis pipeline from fluorescence signals obtained during optical mapping.** (A) A greyscale snap shot of fluorescence from microtissues at 3.2x magnification (also shown in Fig 1E) during optical mapping. Individual microtissues are numbered 1 - 4. (B) Sample membrane voltage (V_m_) traces recorded from the corresponding microtissues in panel A. Note that electrical stimulation did not evoke APs in microtissue #4 (where the fluorescence trace shows a flat line). Fast Fourier transform (FFT) was used to distinguish responsive microtissues from non-responsive microtissues lacking APs. (C) FFT_max_ image. The microtissue (#4) without APs is automatically removed in FFT_max_ (circle). (D) Automated thresholding using Otsu’s thresholding. (E) Blob coloring algorithm to detect individual microtissues. Signals within the same microtissue are averaged to acquire high signal-to-noise ratio and fidelity of data analysis. (F) Automated analysis (of sample trace #3, black) uses the first derivative (dF/dt, red trace) for detecting AP upstroke to calculate duration to 80% recovery (APD_80_, see Figure 1G) or uses the maximum peak of the second derivative (d^2^F/dt^2^, blue trace) for assessing the end of maximum repolarization rate to calculate AP duration APD_MxR_.^1^ APD_MxR_ is useful when motion artifacts or other interference on fluorescence recordings such as fluctuation in water level is suspected to elevate fluorescence level during repolarization and reproducible APD_80_ measurement with low variation is not as reliable. (G) Sample output shows APD_80_ statistics from the 3 excitable individual microtissues.


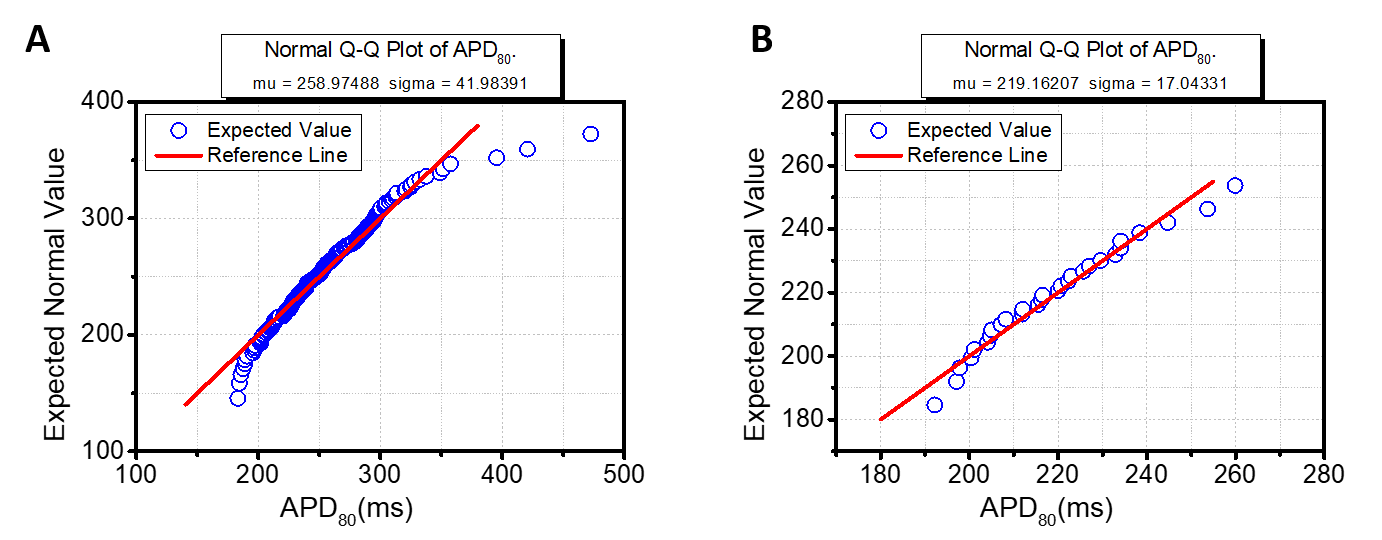


**Supplemental Figure 2. Quantile-Quantile plot of APD data to normal distribution.** APD_80_ metric shows a nearly normal distribution for all data points except at the extremities when compared to the reference line (red) calculated from a normal distribution function for (A) all microtissues from 7 molds from 3 batches and (B) a single batch of 35 microtissues.


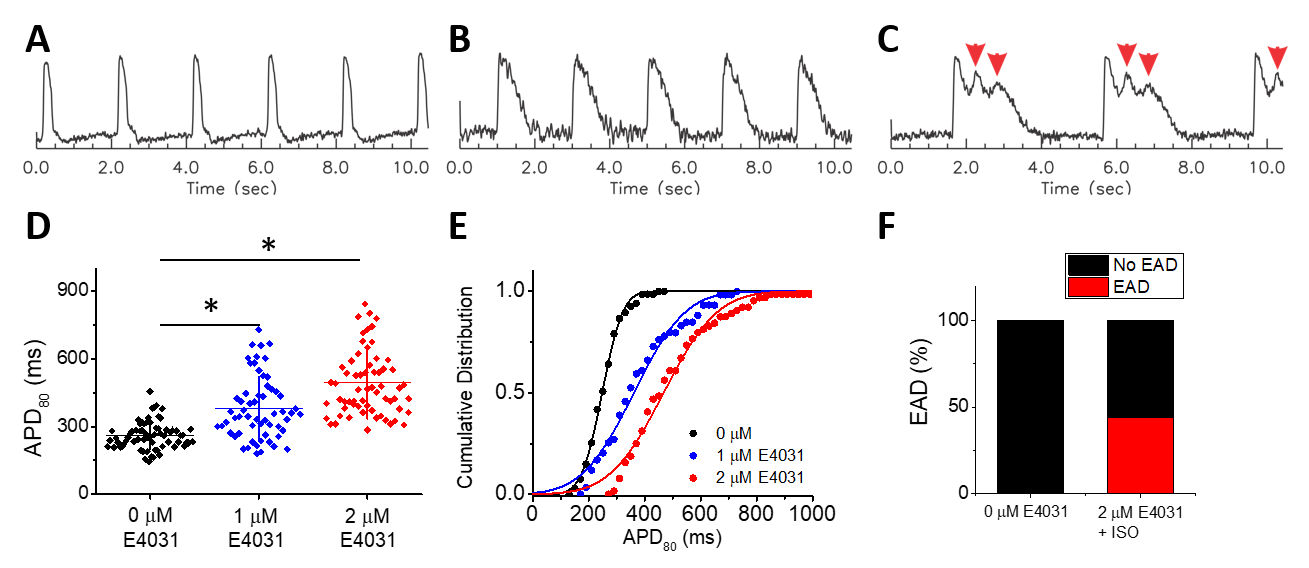


**Supplemental Figure 3. Early after depolarizations in response to hERG channel blocker E4031 and isoproterenol in hiPSC-CM_LP_ with 5% hCF microtissues.** (A-C) Representative V_m_ traces from control (DMSO vehicle, A), 2 µM E4031 exposure (B), and 2 µM E4031 plus 100 nM isoproterenol (ISO, C). APD_80_ are prolonged under 2 µM E4031 and additional beta-adrenergic stimulation triggers early afterdepolarizations (EADs, red arrows). ISO alone does not evoke EADs (see Supplemental Figure S4A). (D, E) Scatter plot (D) and cumulative distribution of APD_80_ (E) show dose-dependent increase of APD_80_ and increased variation with E4031 (n=59-69 microtissues from 2 molds per group). Values shown by lines in (D) are means ± SD. **P* < 0.05 vs. 0 μM. (F) Quantification of EAD incidence before (0 mM) and after (2 µM) E4031 in the presence of 100 nM ISO.


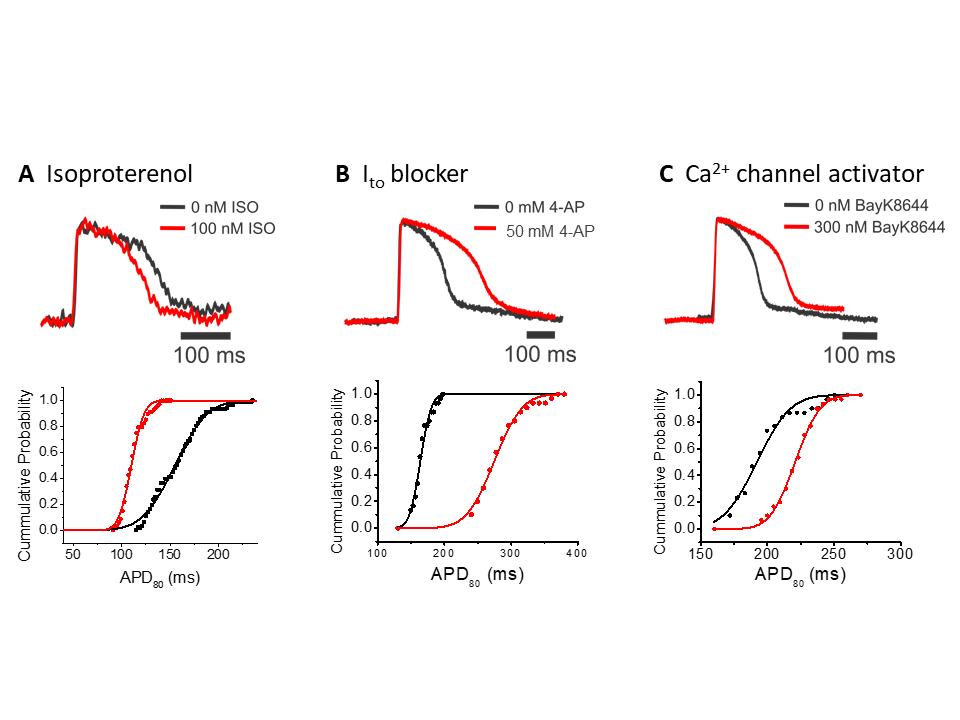


**Supplemental Figure 4. Physiological responses to isoproterenol (ISO), 4-AP, and BayK8644** **in hiPSC-CM_LP_ with 5% hCF microtissues.** Representative traces of (top) and cumulative probability plots for APD_80_ (bottom) show relative changes in response to indicated drugs before and after acute exposure. (A) Beta-adrenergic stimulation with 100 nM ISO shortened APD_80_ (red) (166.6 ± 17.7 vs. 120.1 ± 10.5 after ISO, *P* = 8.91×10^-19^, n=29 microtissues). No early afterdepolarization (EAD) was observed. (B) Blockade of the transient outward potassium current (I_to_) with 4-Aminopyridine (4-AP) prolonged APD_80_ (red) (169.3 ± 14.7 vs. 288.7 ± 33.9 ms, *P* = 2.62×10^-26^, n=35 microtissues). (C) L-type Ca^2+^ channel stimulation with 300 nM BayK8644 prolonged APD_80_ (red) (199.5 ± 20.3 vs. 225.8 ± 14.6 ms, 1.15×10^-9^, n=35 microtissues).


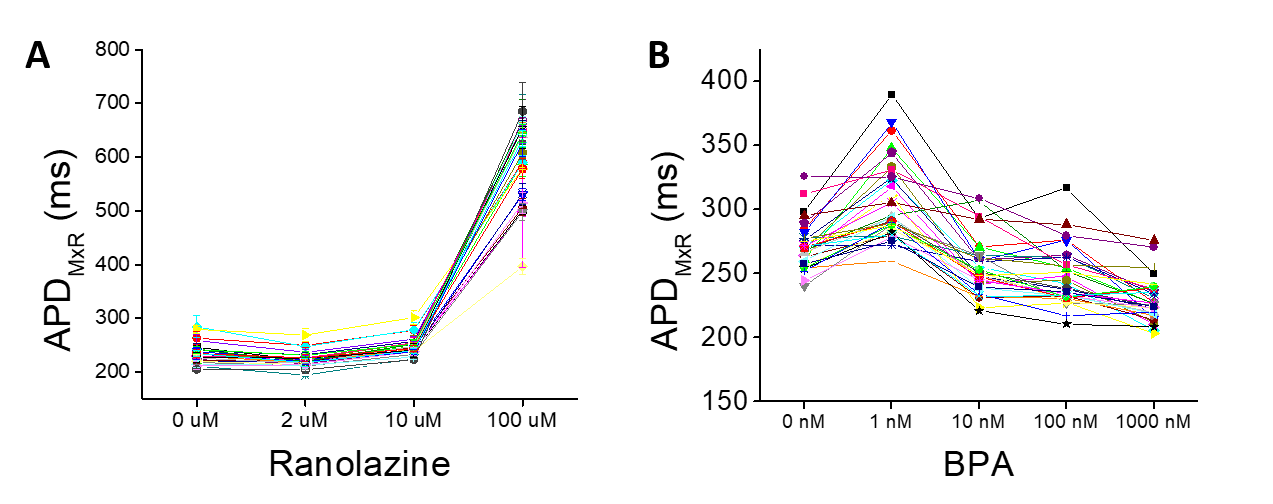


**Supplemental Figure 5. Tracking individual microtissue dose responses of Ranolazine and BPA.** Dose response curves for APD_MxR_ with increasing concentrations of (A) ranolazine (n=34 microtissues; shown in heatmap of Figure 5B) and (B) BPA (n=34 microtissues; heatmap shown in Figure 6B). The lines depict dose-dependent APD_MxR_ changes of individual microtissues. The analysis of individual microtissues before and after drug exposure at different concentrations using paired t-tests increases statistical power.

**
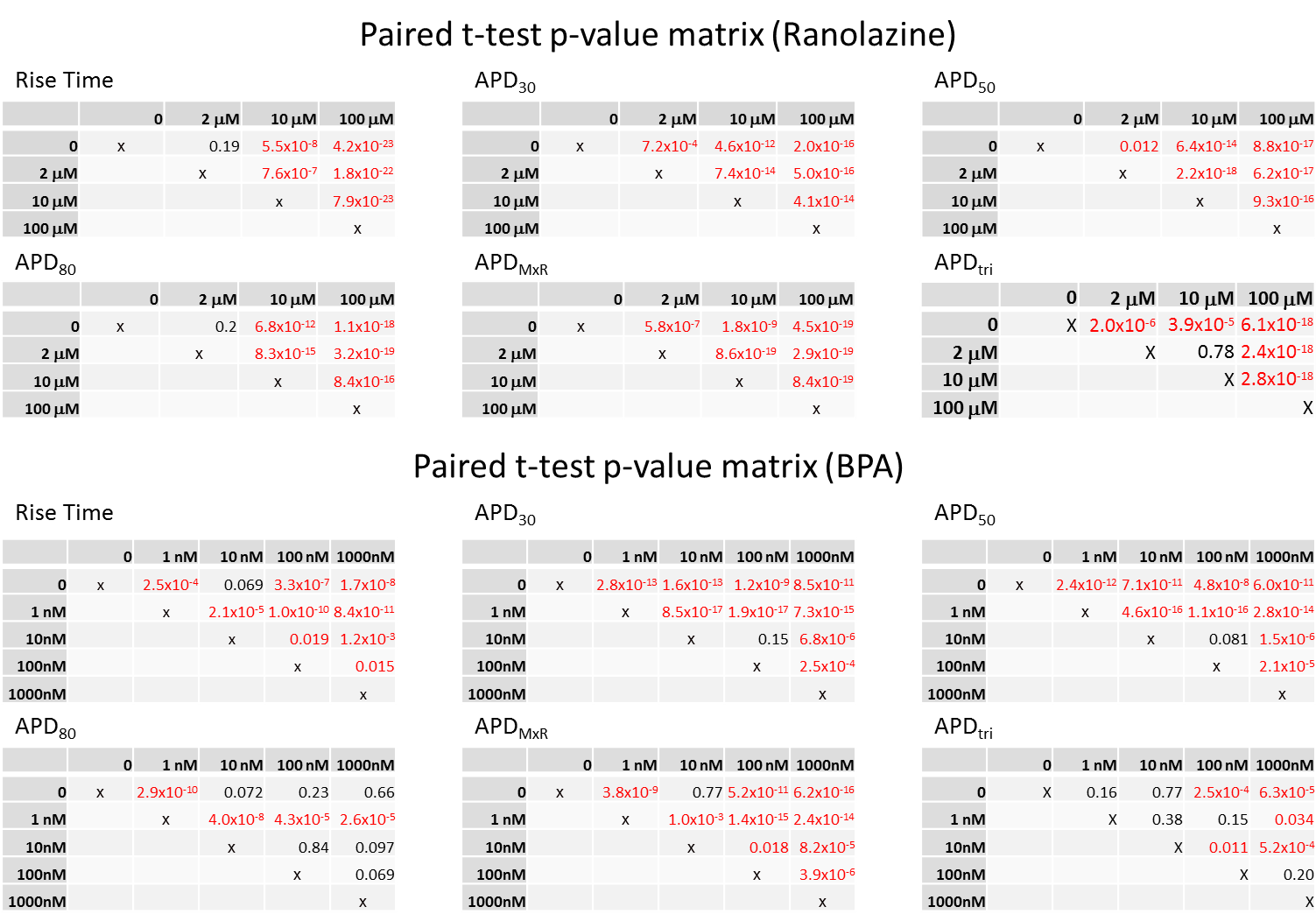
**

**Supplemental Table 1.** *P-*values from paired t-tests of data depicted in Figure 5C for ranolazine (top); Figure 6B for one experiment with BPA (up to 1 μM, bottom). Red font indicates *P*<0.05.


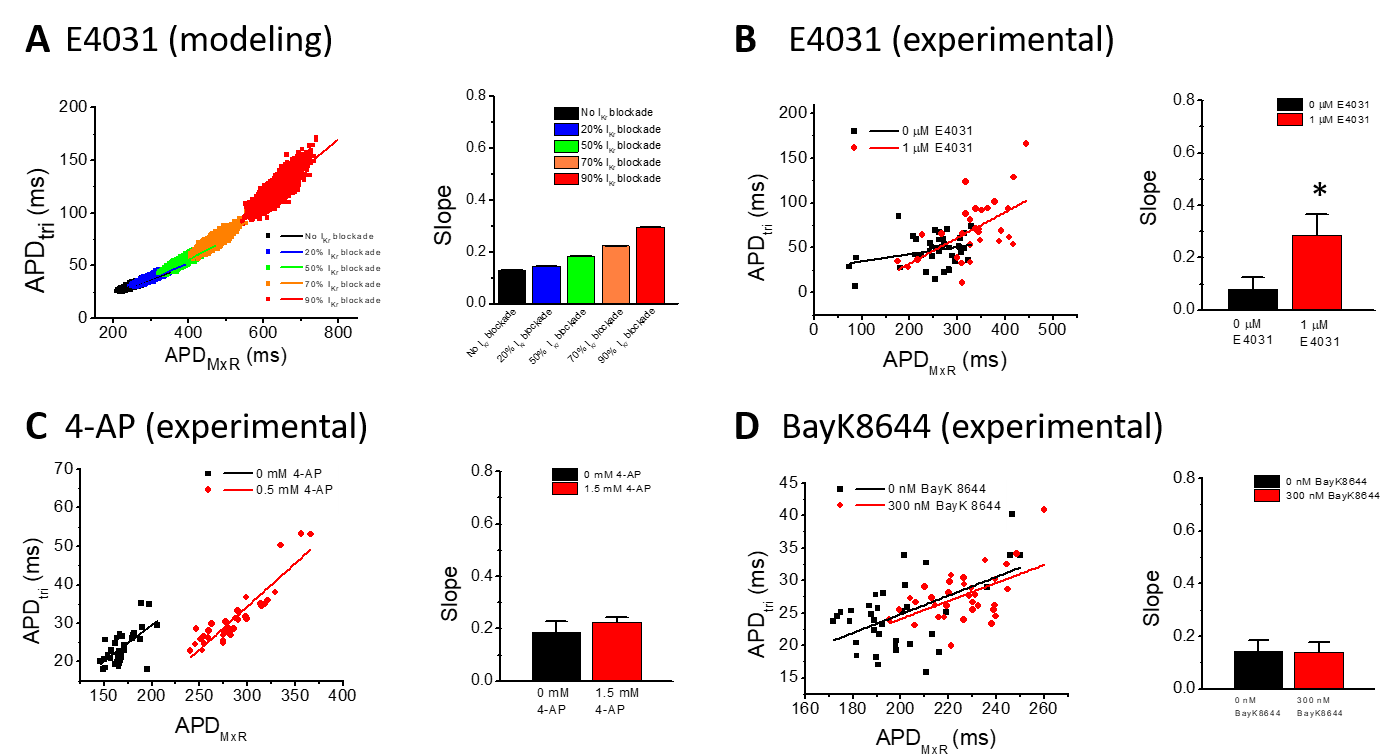


**Supplemental Figure 6. APD_tri_ metric as an indicator of hERG channel blockade.** The relationship between APD prolongation and APD triangulation (APD_tri_) can be used to infer hERG channel blockade. We tested 3 major ion channel modulators that can prolong APD during the early phase (I_to_, phase 1; with 4-AP), plateau phase (I_Ca_, phase 2; with BayK8644), and repolarization (I_Kr_, phase 3; with E4031) of the AP. Since I_Kr_ impacts the later phase of AP repolarization (phase 3), I_Kr_ blockade delays phase 3 repolarization to increase APD_tri_. (A) Computer modeling to study the impact of I_Kr_ blockade on APD_tri_ using the O’Hara human myocyte model.^2^ The conductances of I_Na_, I_to_, I_Ca,_ I_Kr_, I_Ks_ were varied to have a Gaussian distribution of APDs in 10,000 cells (original parameters in O’Hara and Rudy^2^ ± 20%). APD_tri_ changes under I_Kr_ block were investigated. Increase of I_Kr_ blockade (from black to blue, green, orange and red) increases APD_tri_ as APD_MxR_ increases (plot, left) and steepens the slope of APD_tri_ versus APD_MxR_ (bar graph, right). (B-D) Experimental results show that the increase of APD_tri_ is unique to I_Kr_ blockade. (B) The slope of APD_tri_ versus APD_MxR_ steepened in response to 1µM E4031, in agreement with the results from the computer modeling in panel A. The slope significantly increases between 0 and 1 µM E4031 (right, p = 0.011). (C) 0.5 mM 4-AP (I_to_ blockade) did not steepen the slope of APD_tri_ versus APD_MxR_ despite APD prolongation (p = 0.199). (D) 300 nM BayK8644 (I_Ca_ agonist) did not steepen APD_tri_ versus APD_MxR_ despite APD prolongation (p = 0.539).

Dose-dependent mean differences of AP metrics (Ranolazine)

| Ranolazine | 2 μM | 10 μM | 100 μM |
| --- | --- | --- | --- |
| Rise Time | -0.35093 | 0.03604 | 7.77581 |
| APD_30_ | 4.39627 | 16.6689 | 53.5831 |
| APD_50_ | -3.46581 | 14.7128 | 101.51 |
| APD_80_ | 3.43512 | 64.7965 | 219.988 |
| APD_MxR_ | -10.5228 | 6.64536 | 307.389 |
| APD_tri_ | -3.5293 | 2.56326 | 198.626 |

Dose-dependent mean differences of AP metrics (BPA)

| BPA | 1 nM | 10 nM | 100 nM |
| --- | --- | --- | --- |
| Rise Time | -0.12072 | -1.53018 | -2.27589 |
| APD_30_ | 19.687 | -10.3786 | -16.5818 |
| APD_50_ | 16.8487 | -15.1815 | -21.6624 |
| APD_80_ | 22.9907 | 1.23495 | 0.76422 |
| APD_MxR_ | 10.2523 | -19.7025 | -31.285 |
| APD_tri_ | -6.57858 | -4.48536 | -9.56911 |

**Supplemental Table 2.** Mean dose-response difference of AP metrics. Red and blue fonts indicate that AP metrics increased and decreased, respectively, by the paired t-test in Figure 7.

| Metric (units) | Ion channels involved | Ion currents |
| --- | --- | --- |
| Excitability (%) and stimulation time delay | Na channels (Na_v_1.5, 1.1, others) Inward rectifier K^+^  channel (Kir2.1) | I_Na_ I_K1_ |
| Rise time (ms) | Na channel (Na_v_1.5) | I_Na_ |
| APD30 (ms) | Late Na channels (Na_v_1.1, others) Ca channels (Ca_v_1.2) K channels (Kv4.2/4.3, Kv1.4) | I_Na,late_ I_CaL_  I_to_ |
| APD50 (ms) | Late Na channels (Na_v_1.1, others) Ca channels (Ca_v_1.2) K channels (hERG, KvLQT, Kv4.2/4.3, Kv1.4) | I_Na,late_ I_CaL_ I_to_, I_Kr_, I_Ks_ |
| APD80 (ms) | K channels (hERG, KvLQT, Kir2.1) | I_Kr_, I_Ks,_ I_K1_ |
| APDtri (ms) =APD80-APD30 | Late Na channels (Na_v_1.1, others) Ca channels (Ca_v_1.2) K channels (hERG, KvLQT, Kv4.2/4.3, Kv1.4, Kir2.1) | I_Na,late_ I_CaL_ I_to_, I_Kr_, I_Ks_, I_K1_ |
| EADs (count/AP) | K channels (hERG, KvLQT ) late Na channel (Nav1.5) | I_Kr,_ I_Ks_ I_Na,late_ |

**Supplemental Table 3.** Quantitative metrics and physiological targets of the AP.

**
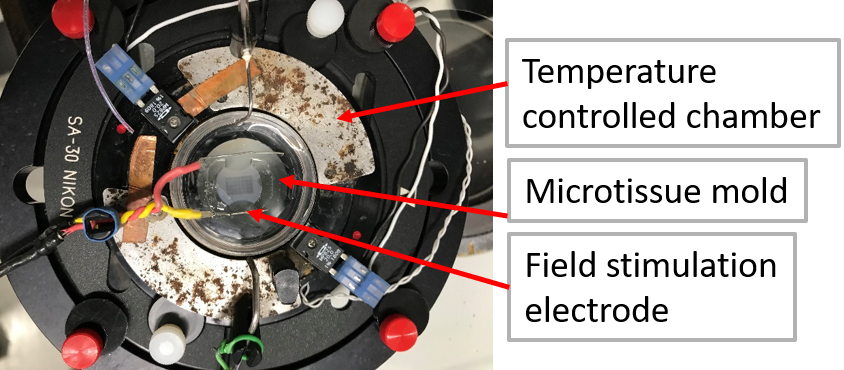
**

**Supplemental Figure 7.** Stimulation electrode set-up during optical mapping image acquisition.

**Movie S1.** Beating human cardiac microtissue comprised of hiPSC-CMs and 5% hCFs imaged after 1 day in 3D culture while paced at 1 Hz.

**Movie S2.** Optical mapping of a human cardiac microtissue (left) and the corresponding voltage trace (right) under control conditions (DMSO as vehicle) while paced at 0.5 Hz.

**Movie S3.** Optical mapping of 2 human cardiac microtissues (left) and the corresponding voltage traces (right) under 2 μM E4031 showing APD prolongation and distinct EADs while paced at 0.5 Hz.

**Supplemental Material References:**

1. Efimov IR, Huang DT, Rendt JM, Salama G. Optical mapping of repolarization and refractoriness from intact hearts. *Circulation* 1994;**90**:1469-1480.

2. O'Hara T, Virag L, Varro A, Rudy Y. Simulation of the undiseased human cardiac ventricular action potential: model formulation and experimental validation. *PLoS Comput Biol* 2011;**7**:e1002061.
